# Supplementary material for: Silver-Catalyzed Decarboxylative Acylation of Isocyanides Accesses to α-Ketoamides with Air as a Sole Oxidant
Source: Molecules. 2023 Jul 11;28(14):5342. doi: 10.3390/molecules28145342 (PMC10383724; doi:10.3390/molecules28145342)
Supplement: Supplementary file 1 [file molecules-28-05342-s001.zip › molecules-2490065-supplementary.pdf]

Silver-Catalyzed Decarboxylative Acylation of Isocyanides Accesses to α-ketoamides with Air as a Sole Oxidant

Jia Xu [†], Xue Li [†], Xing-Yu Chen, Yu-Ting He, Jie Lei, Zhong-Zhu Chen * and Zhi-Gang Xu *

College of Pharmacy, National & Local Joint Engineering Research Center of Targeted and Innovative Therapeutics, IATTI, Chongqing University of Arts and Sciences, Chongqing 402160, China; xujia_0907@163.com (J.X.); lixue91jlsp@sina.com (X.L.); faith0909@163.com (X.-Y.C.); jjjxu_92@126.com (Y.-T.H.); jlei0916@163.com (J.L.)

* Correspondence: 18883138277@163.com (Z.-Z.C.); xzg@cqwu.edu.cn (Z.-G.X.)

[†] These authors contributed equally to this work.

Table of Contents **Page**

Control experiments………………………………………………S2

X-ray structure of **4**………………………………………………S2

NMR Characterization Figures of Products…………………………S3-S31

Control experiments

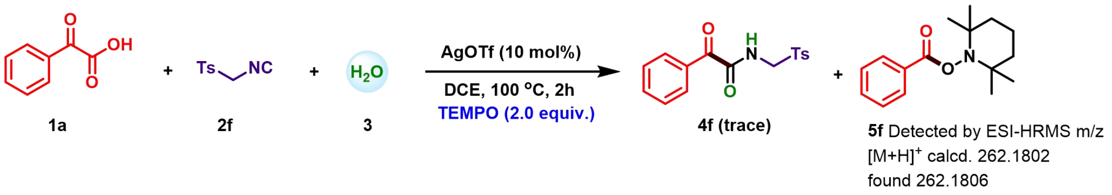

Phenylglyoxylic acid (0.3 mmol, 45 mg, 1.0 equiv.), toluenesulfonylmethyl isocyanide (0.3 mmol, 68 mg, 1.0 equiv.), water (0.6 mmol, 11 μL, 2.0 equiv.), and AgOTf (10 mol%, 8 mg) and TEMPO (0.6 mmol, 94 mg, 2.0 equiv.) were dissolved in DCE (2.0 mL). The reaction mixture was stirred at 100 °C for 2 h in 5mL of microwave vial. After the completion the reaction mixture, the ESI-MS analysis of the crude reaction mixture was carried out. The mass for benzoyl radical trapping product **5f** could be detected. The mass for **5f** is $C_{16}H_{23}NO_2^+$ $[M+H]^+$ calcd. 262.1806, found 262.1806.

Table S1. Control experiments for reagents.

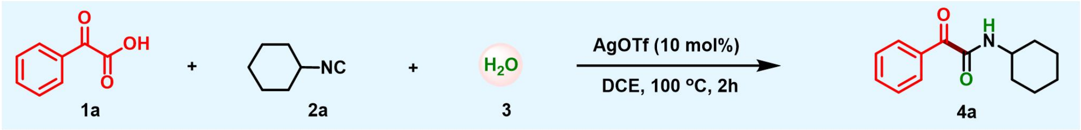

Entry	1a	2a	3	Yield（%）
**1**	**0.3 mmol**	**0.3 mmol**	**0.6 mmol**	**89**
2	0.3 mmol	0.3 mmol	0.9 mmol	67
3	0.3 mmol	0.3 mmol	0.3mmol	73
4	0.6 mmol	0.3 mmol	0.6 mmol	89
5	0.3 mmol	0.6 mmol	0.6 mmol	85

X-ray structure of 4a (CCDC 2267589)

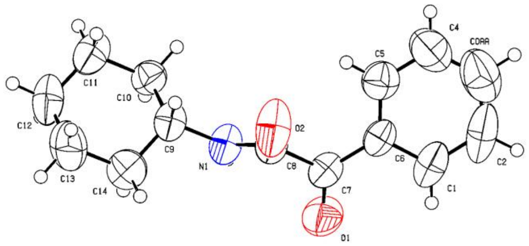

Method: Compound **4a** (20 mg) was dissolved in 10.0 mL 40% EA/Hex at room temperature. The solution was transferred to a 25 mL bottle, and as the solution volatilized, crystal growth can be found.

NMR Characterization Figures of Products 4a-4ac

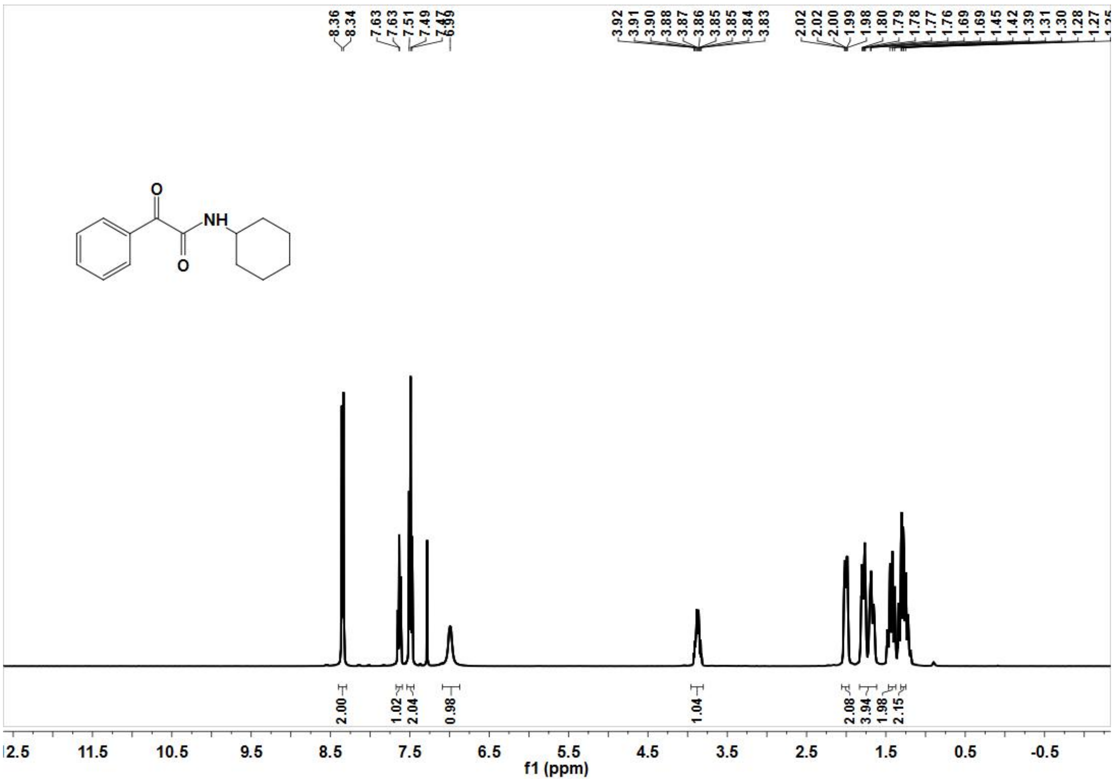

Figure S1. ^{1}H NMR (400 MHz, $CDCl_3$) spectrum of **4a.**

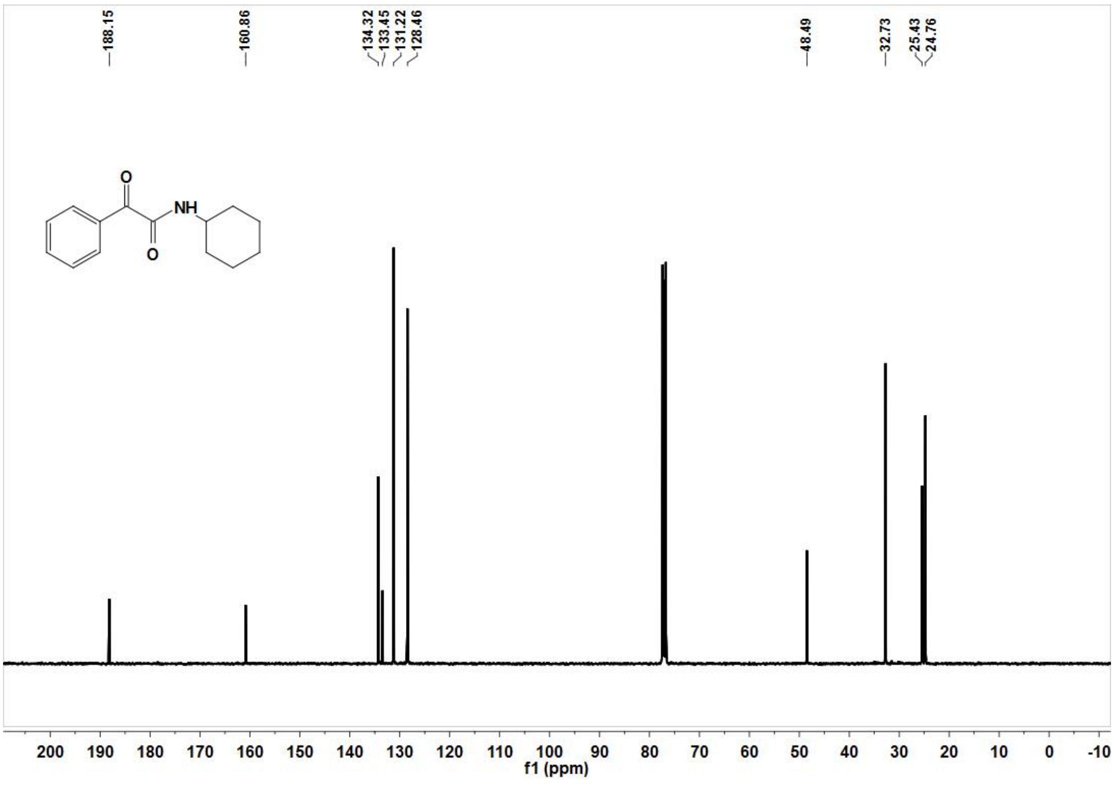

Figure S2. ^{13}C NMR (100 MHz, $CDCl_3$) spectrum of **4a.**

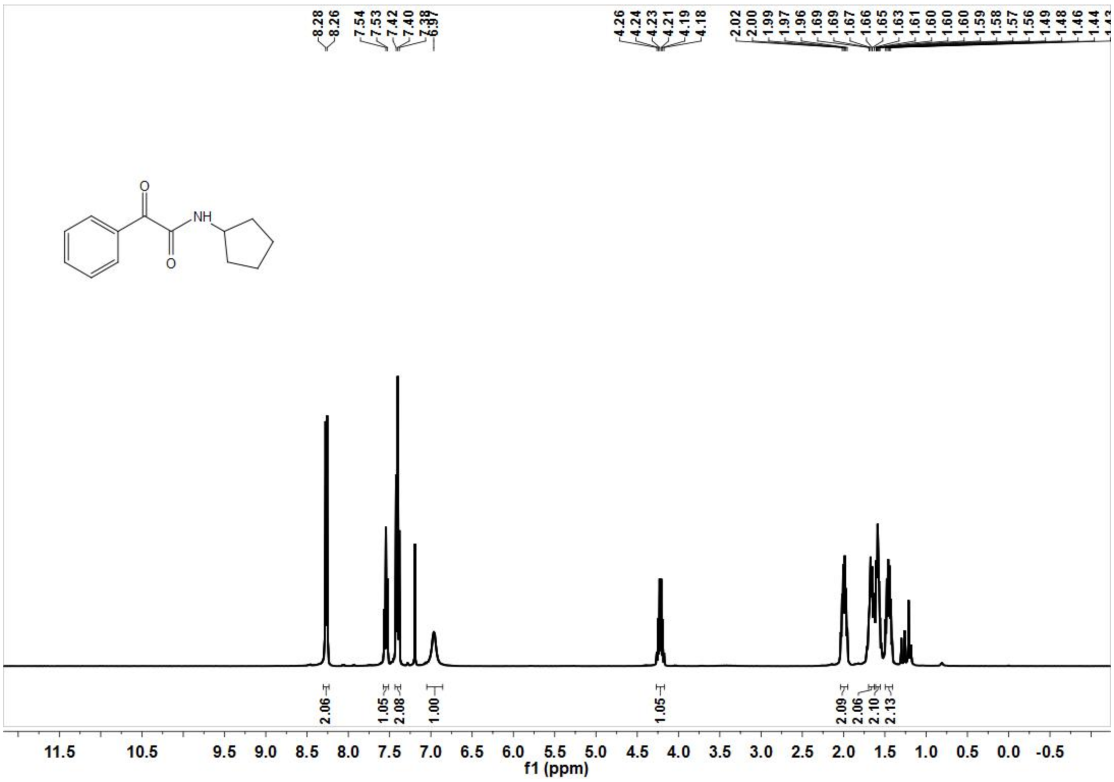

Figure S3. ^{1}H NMR (400 MHz, $CDCl_3$) spectrum of **4b.**

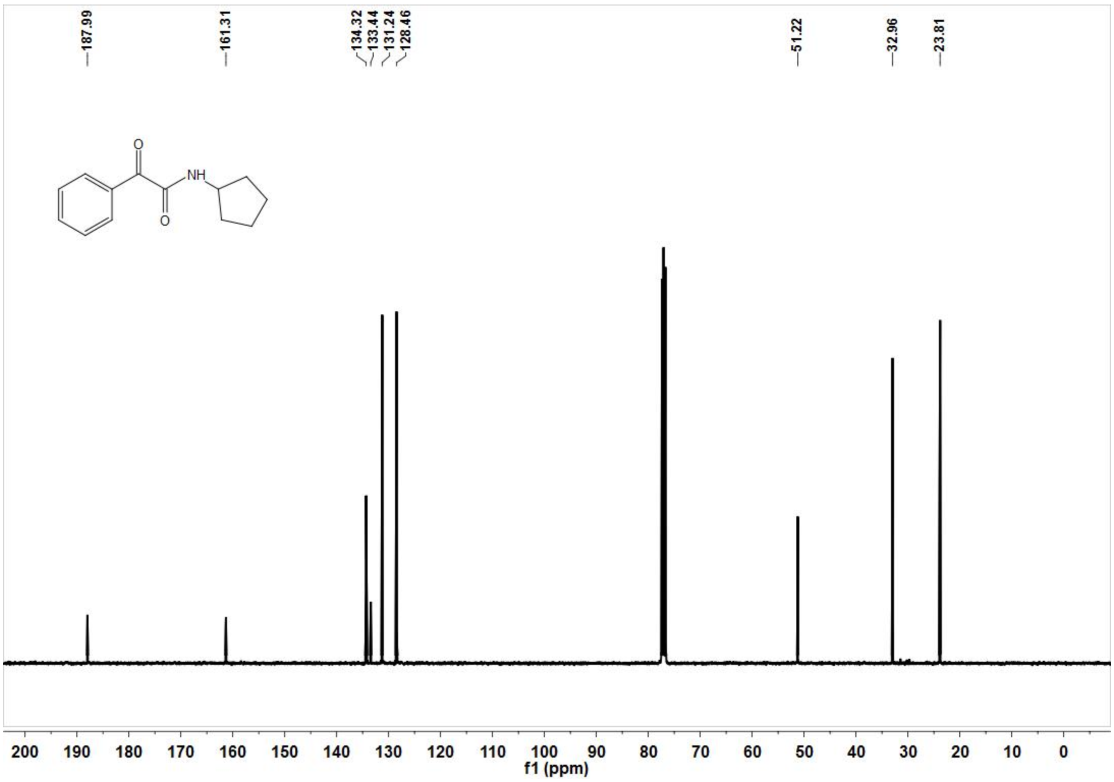

Figure S4. ^{13}C NMR (100 MHz, $CDCl_3$) spectrum of **4b.**

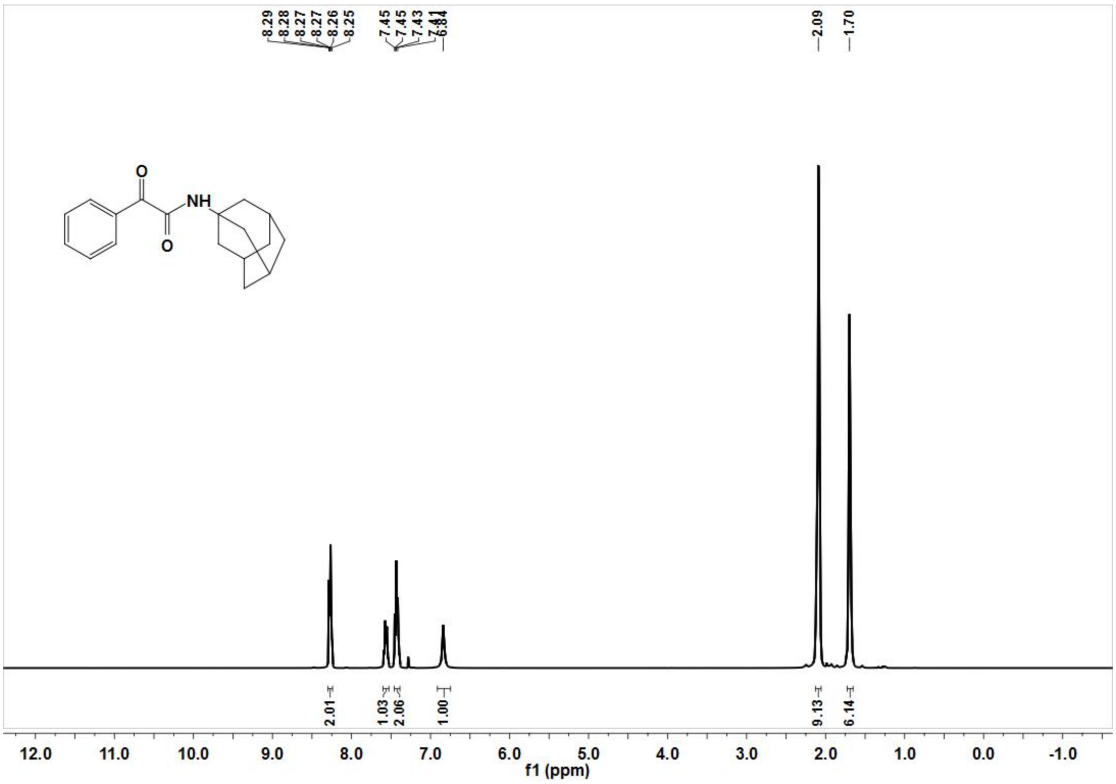

Figure S5. ^{1}H NMR (400 MHz, $CDCl_3$) spectrum of **4c.**

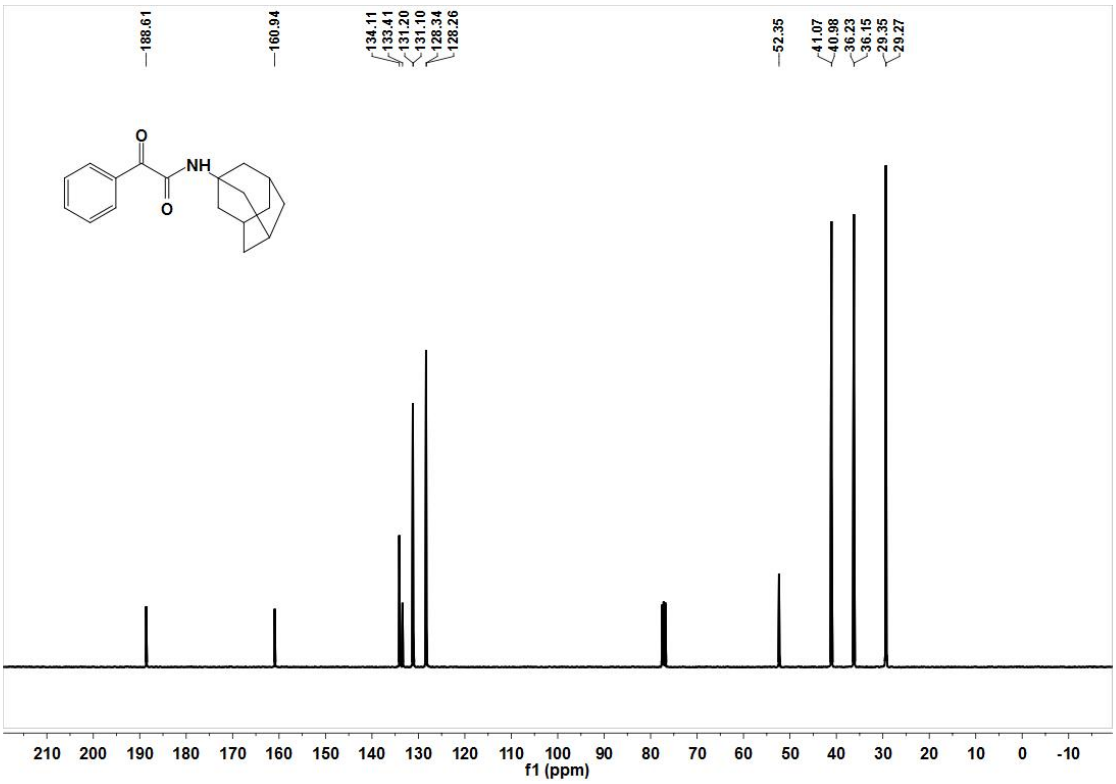

Figure S6. ^{13}C NMR (100 MHz, $CDCl_3$) spectrum of **4c.**

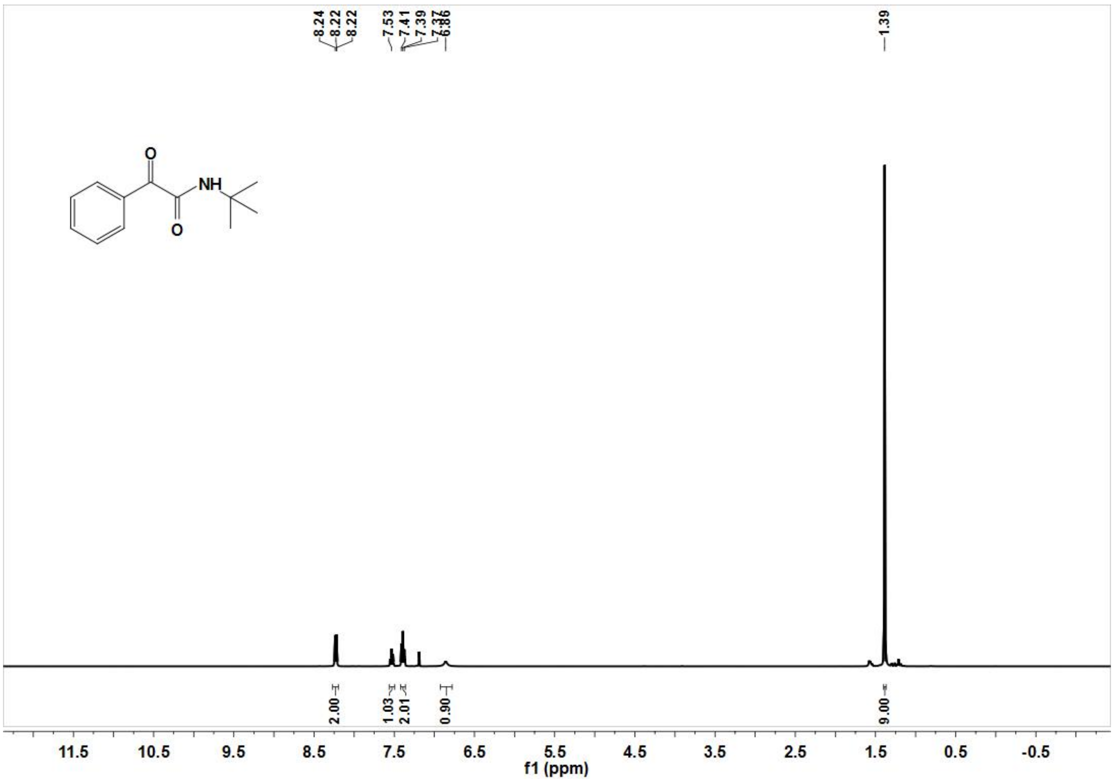

Figure S7. ^{1}H NMR (400 MHz, $CDCl_3$) spectrum of **4d.**

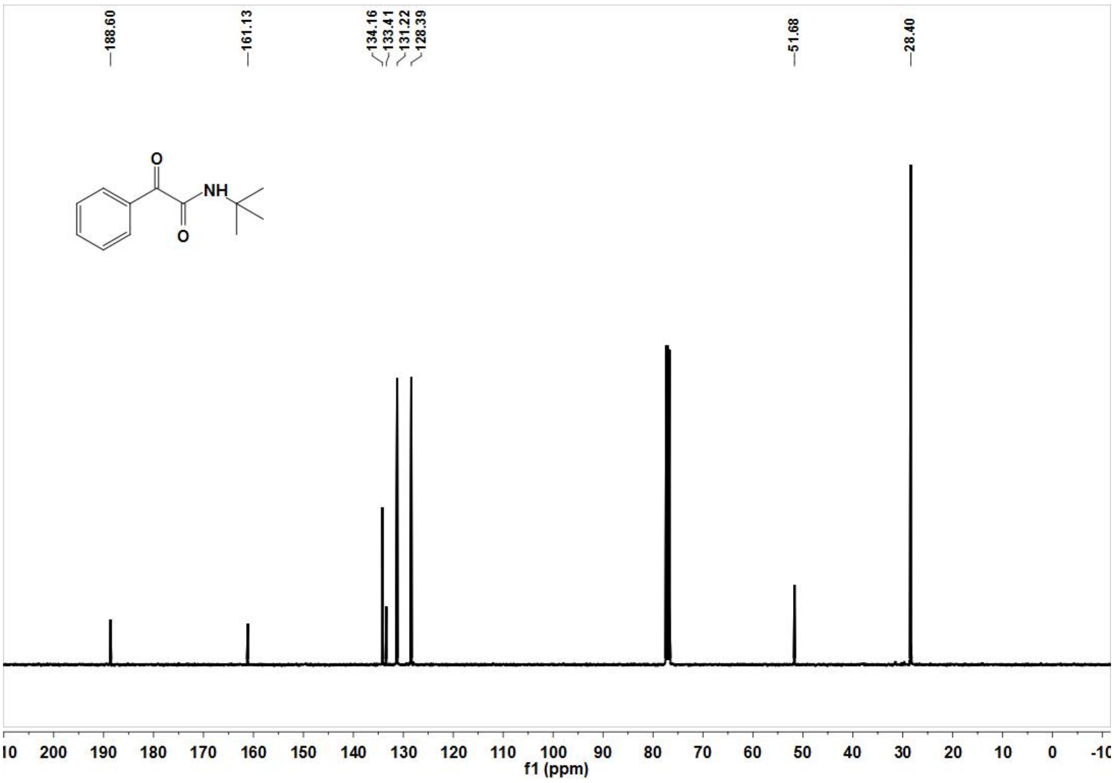

Figure S8. ^{13}C NMR (100 MHz, $CDCl_3$) spectrum of **4d.**

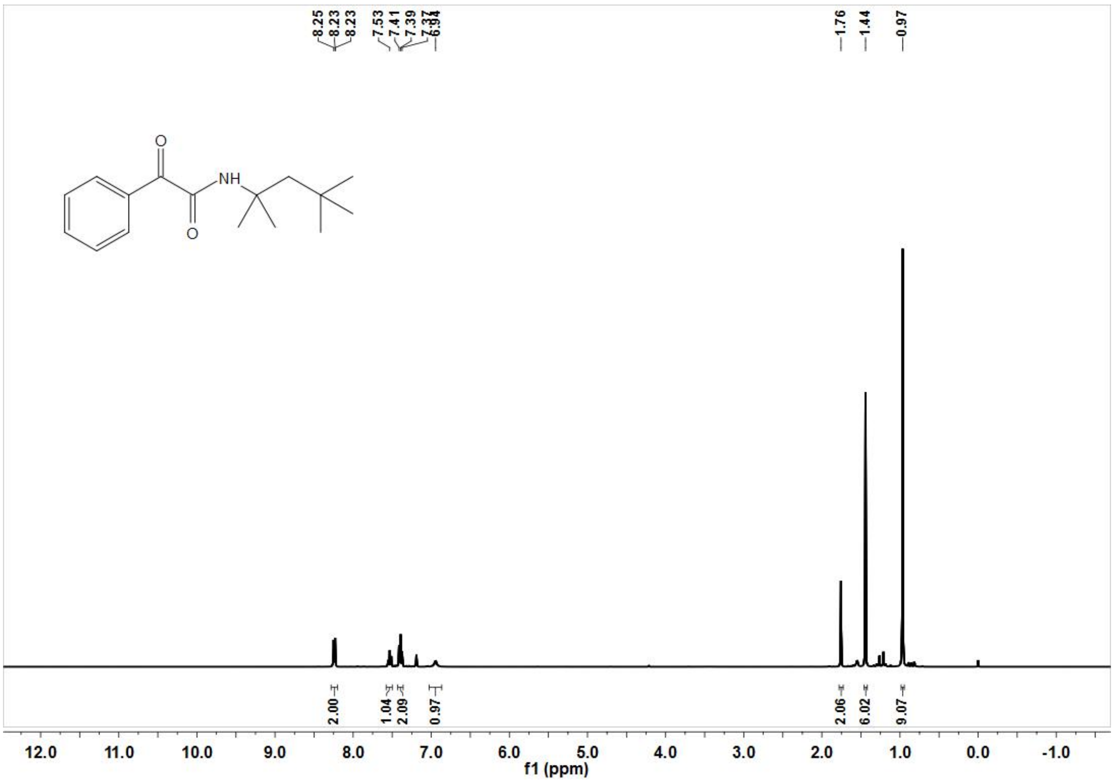

Figure S9. ^{1}H NMR (400 MHz, $CDCl_3$) spectrum of **4e.**

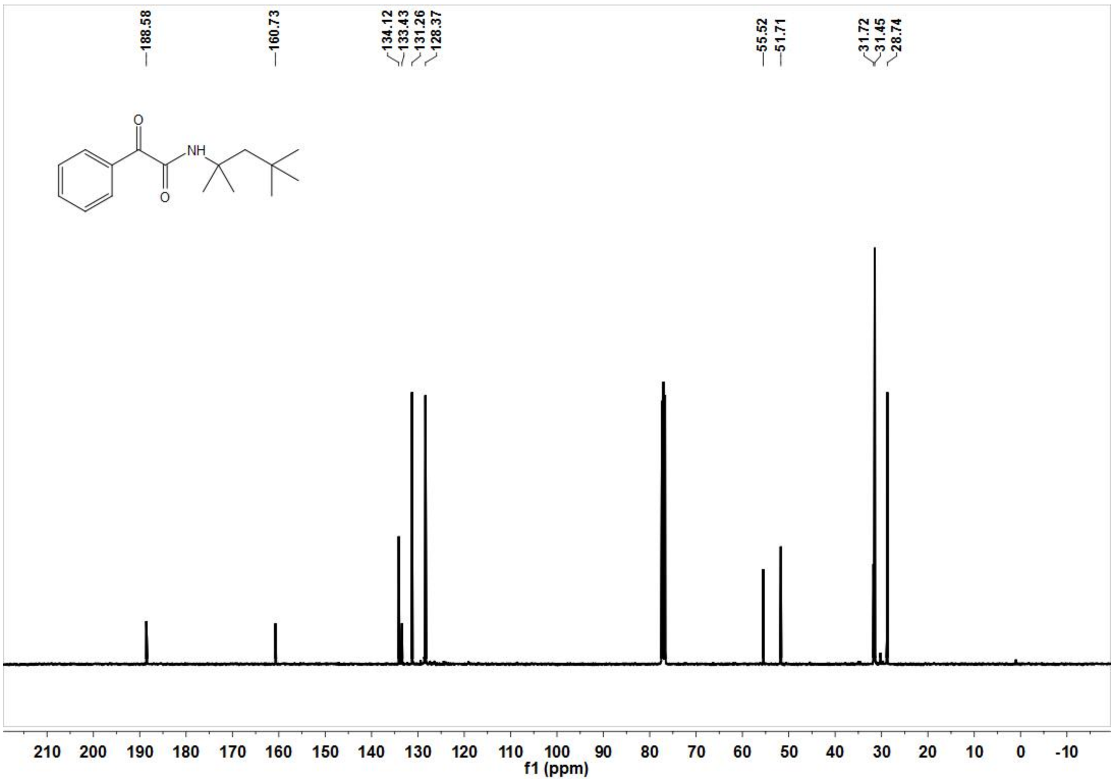

Figure S10. ^{13}C NMR (100 MHz, $CDCl_3$) spectrum of **4e.**

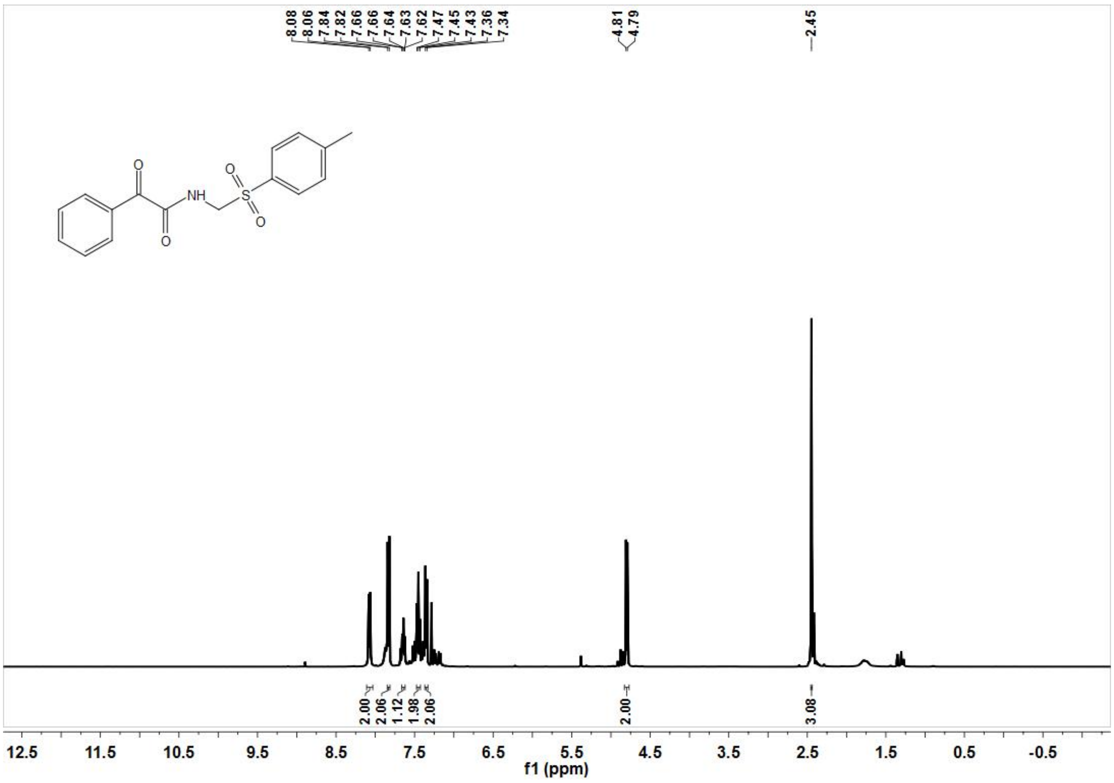

Figure S11. ^{1}H NMR (400 MHz, $CDCl_3$) spectrum of **4f.**

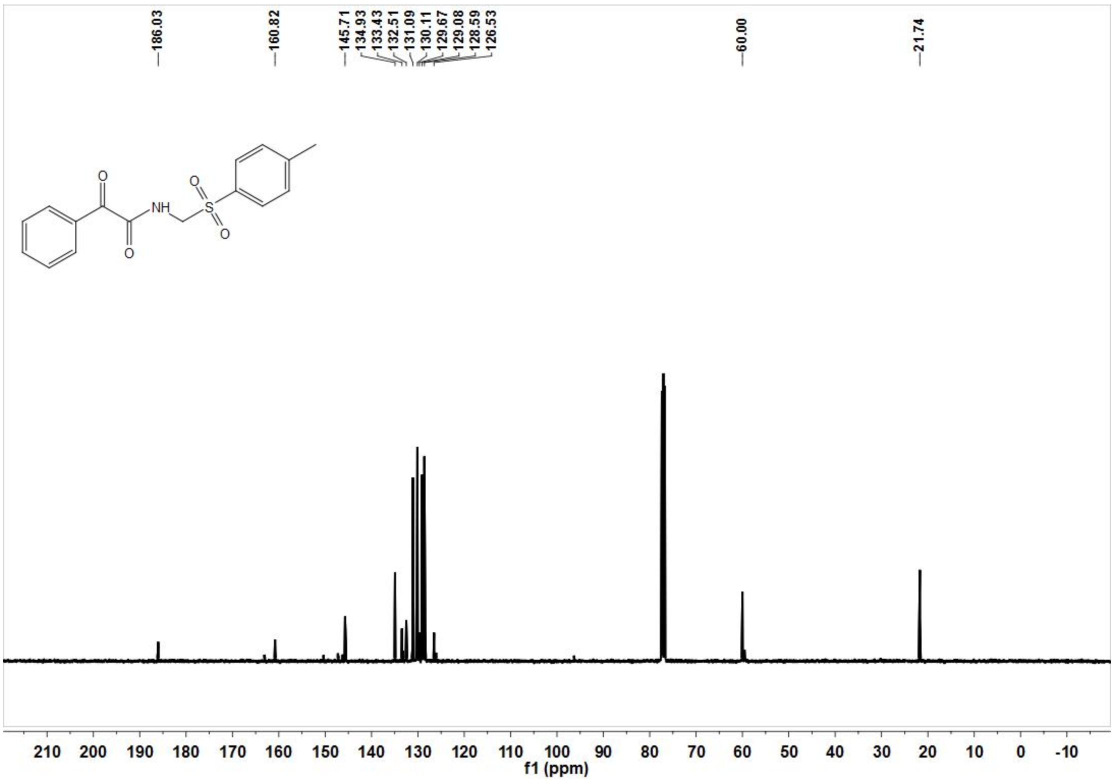

Figure S12. ^{13}C NMR (100 MHz, $CDCl_3$) spectrum of **4f.**

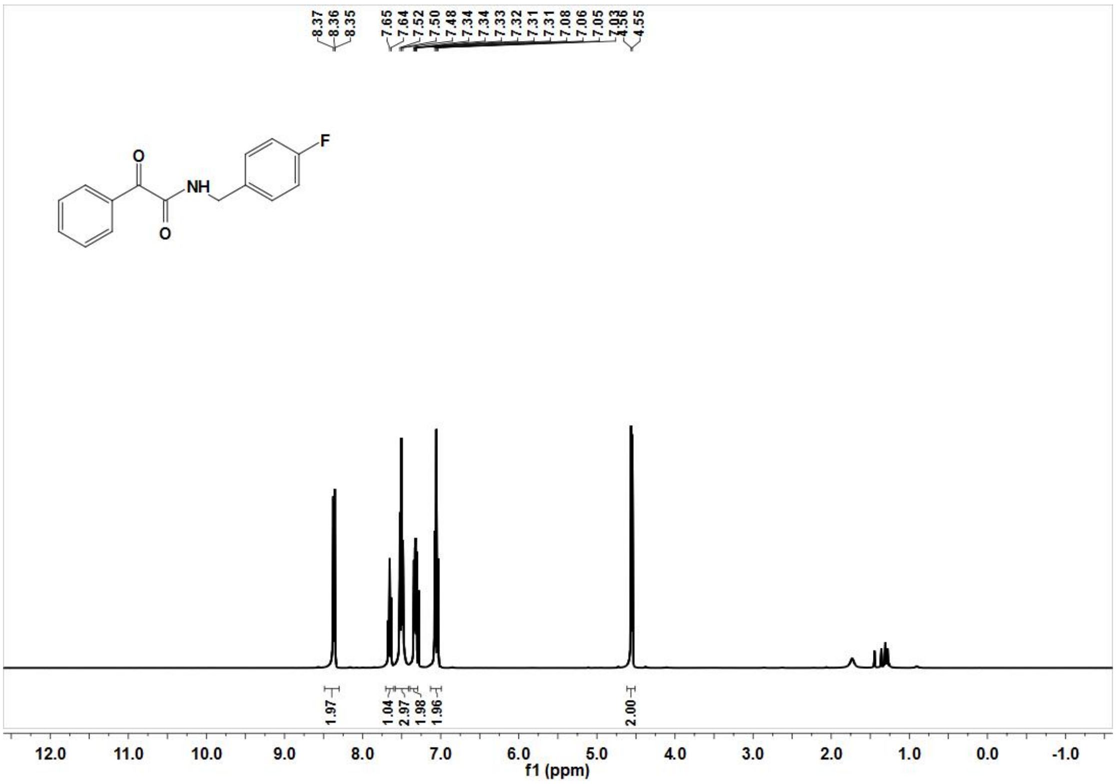

Figure S13. ^{1}H NMR (400 MHz, $CDCl_3$) spectrum of **4g.**

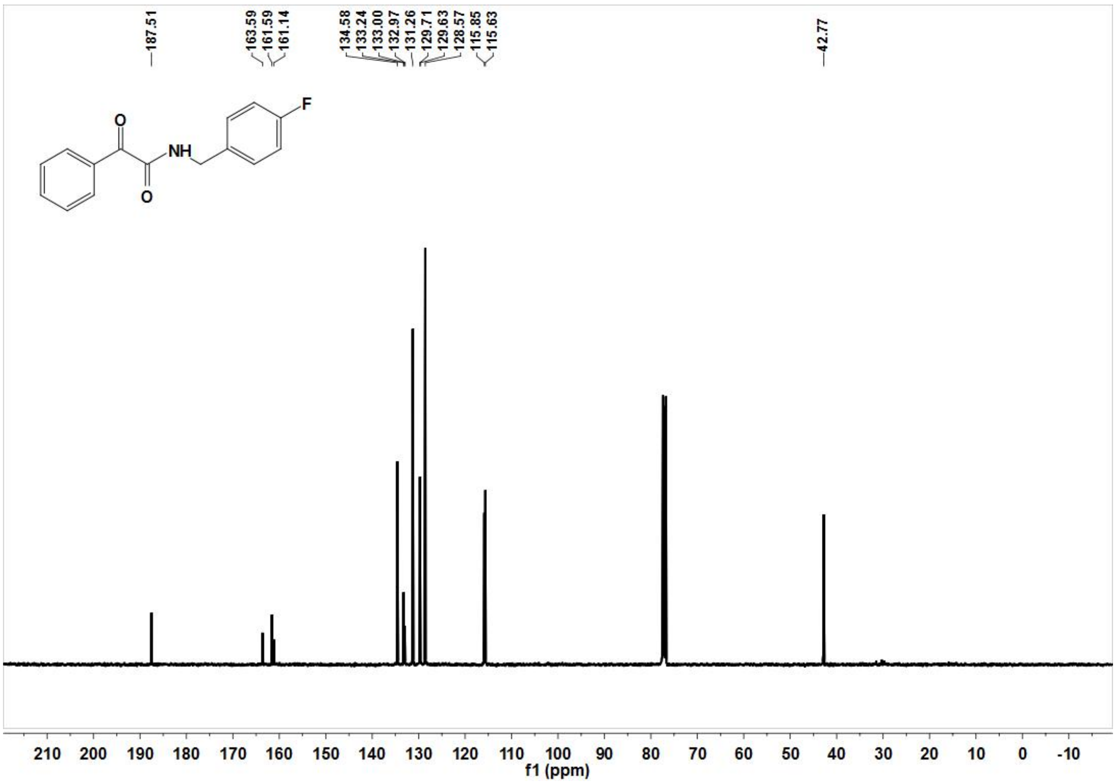

Figure S14. ^{13}C NMR (100 MHz, $CDCl_3$) spectrum of **4g.**

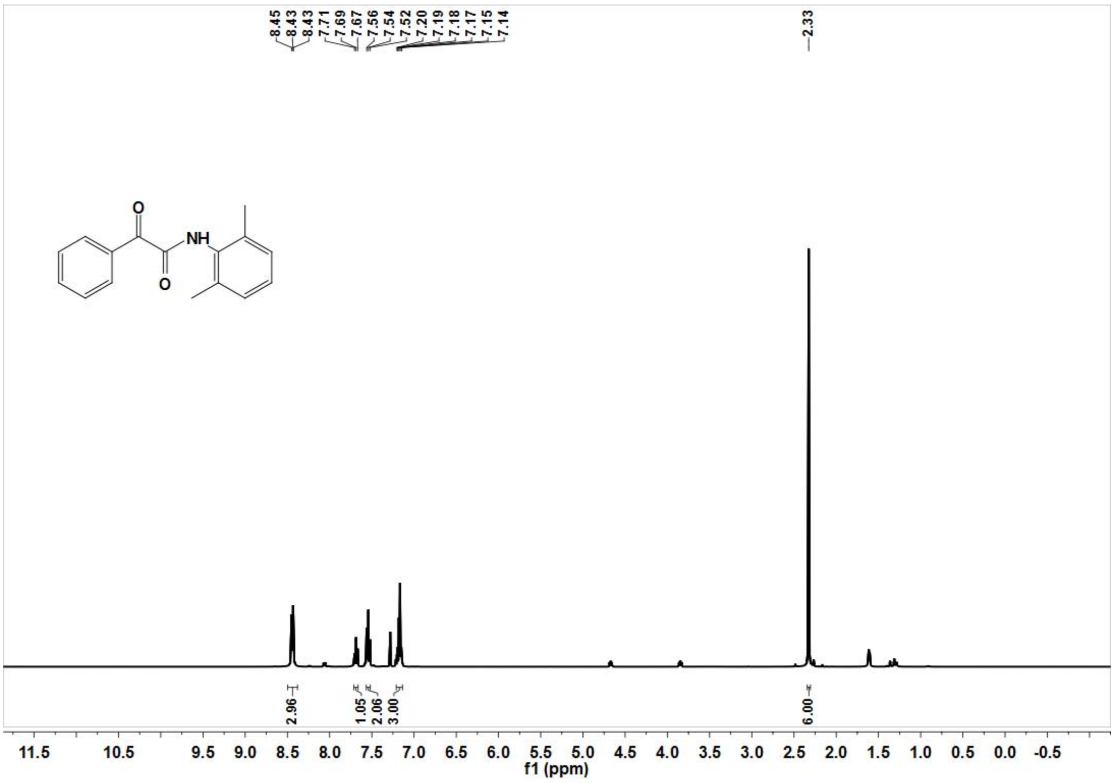

Figure S15. ^{1}H NMR (400 MHz, $CDCl_3$) spectrum of **4h.**

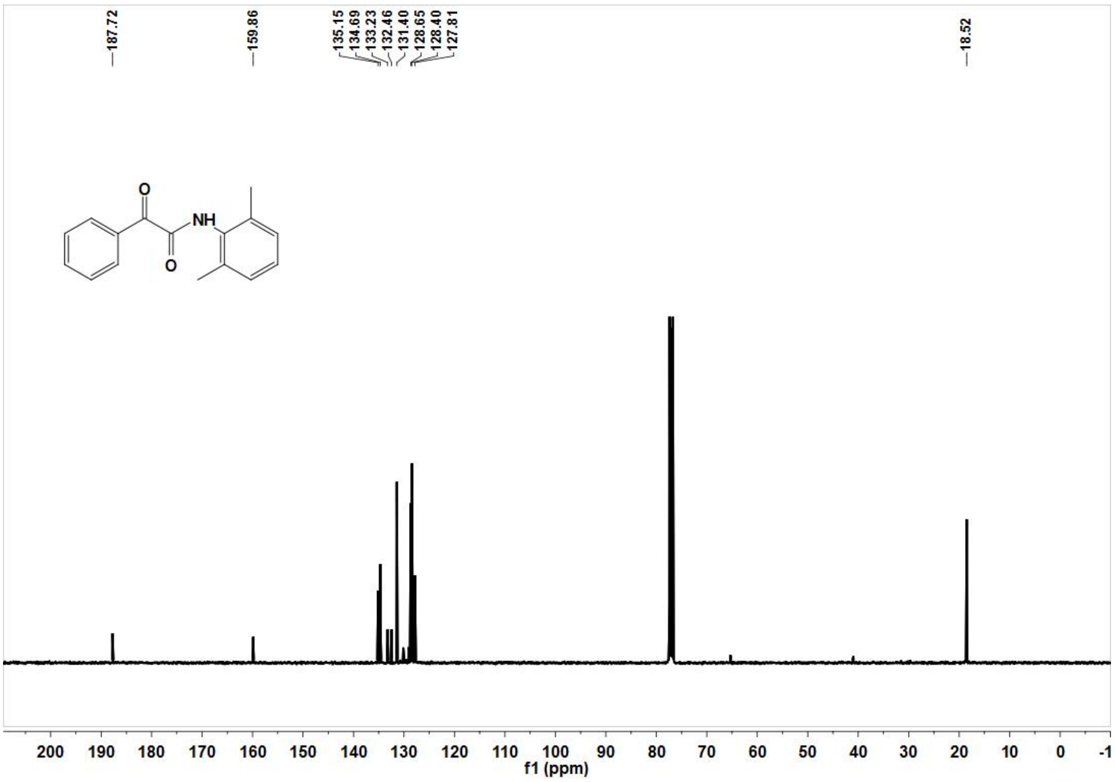

Figure S16. ^{13}C NMR (100 MHz, $CDCl_3$) spectrum of **4h.**

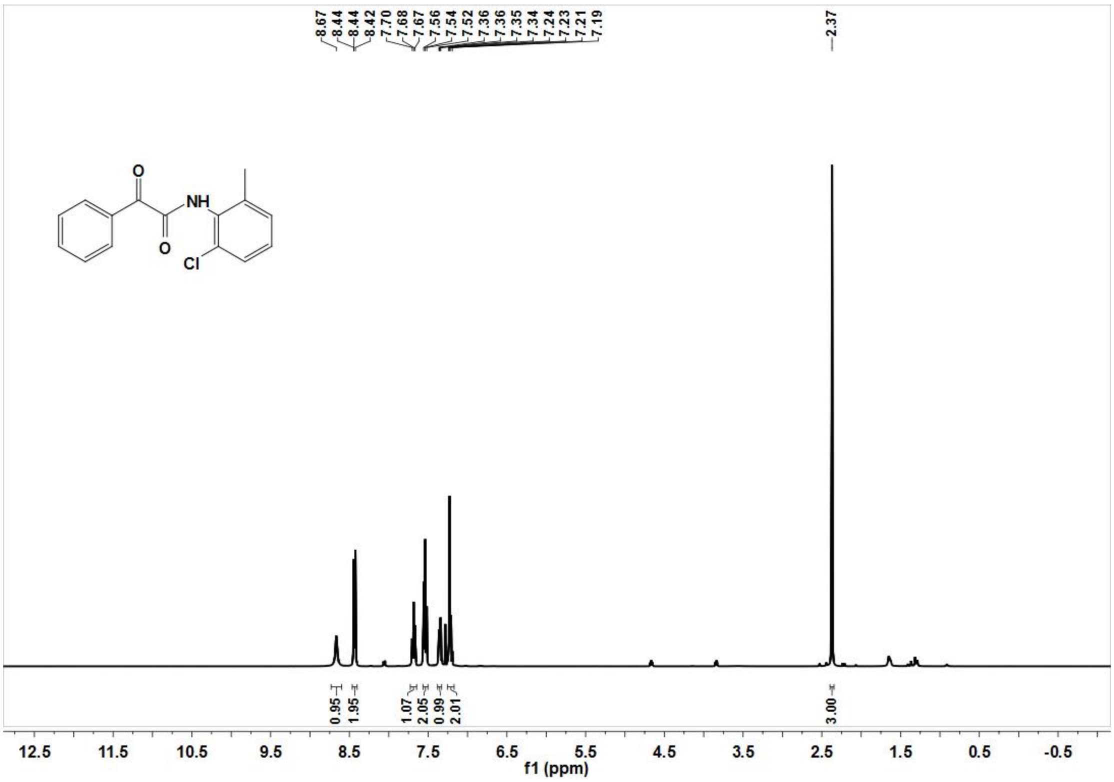

Figure S17. ^{1}H NMR (400 MHz, $CDCl_3$) spectrum of **4i.**

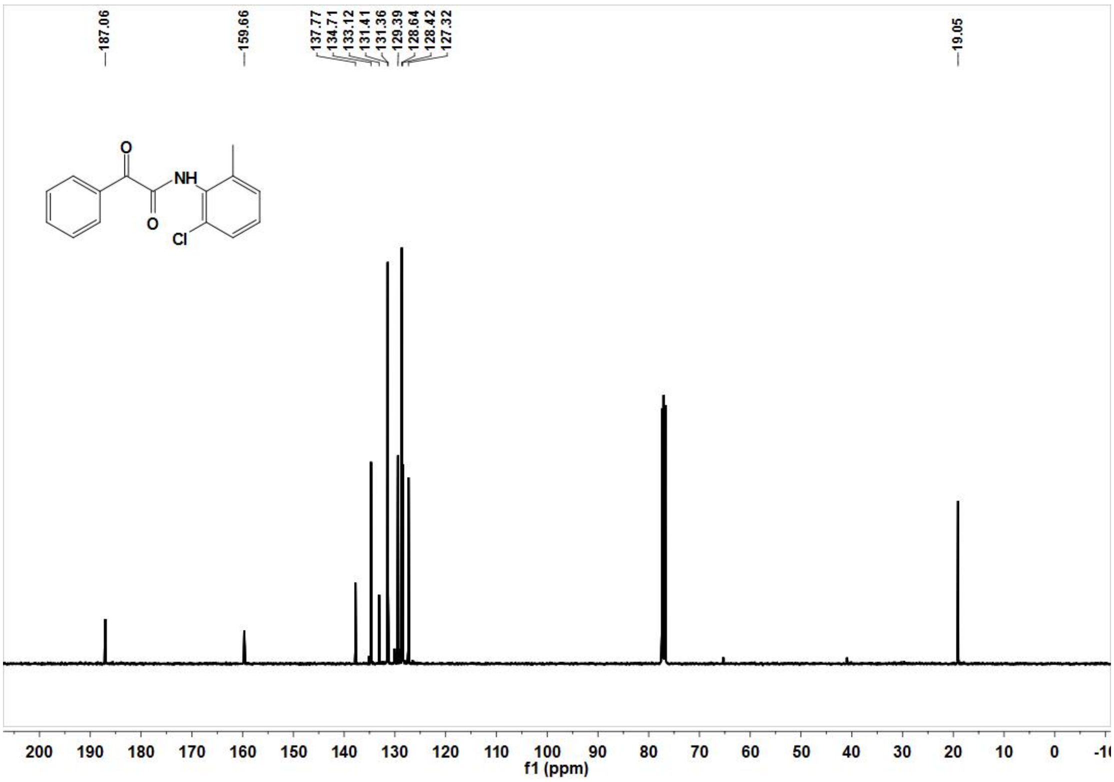

Figure S18. ^{13}C NMR (100 MHz, $CDCl_3$) spectrum of **4i.**

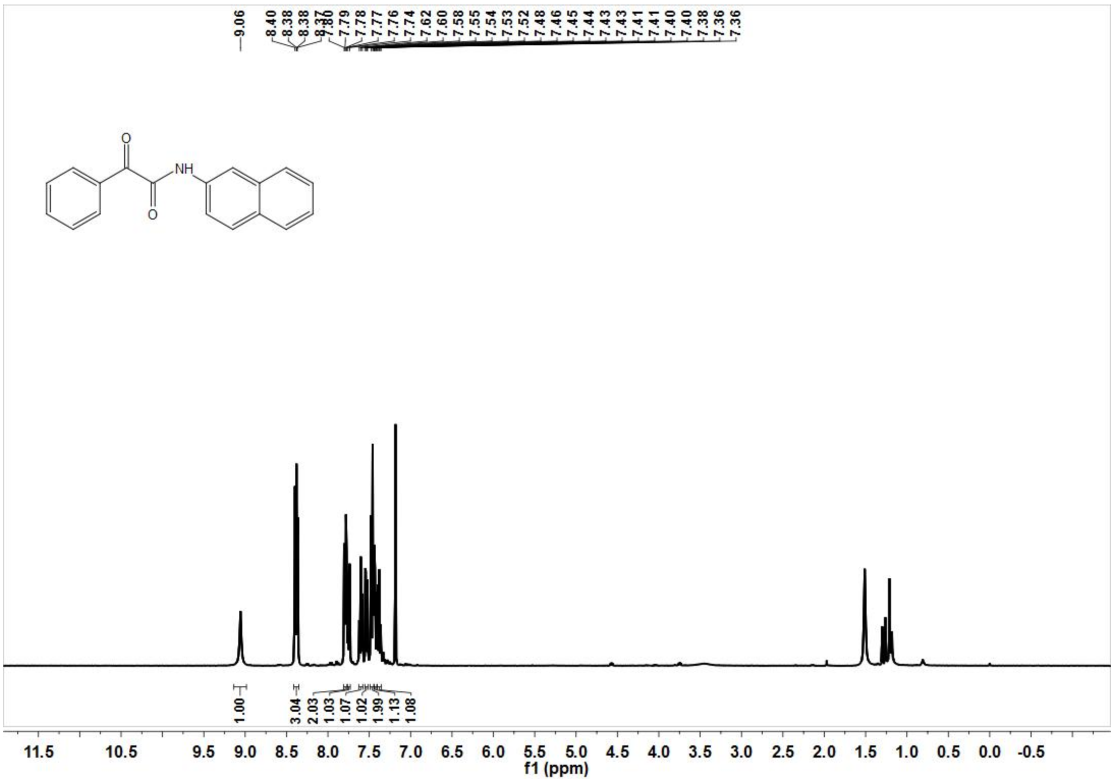

Figure S19. ^{1}H NMR (400 MHz, $CDCl_3$) spectrum of **4j.**

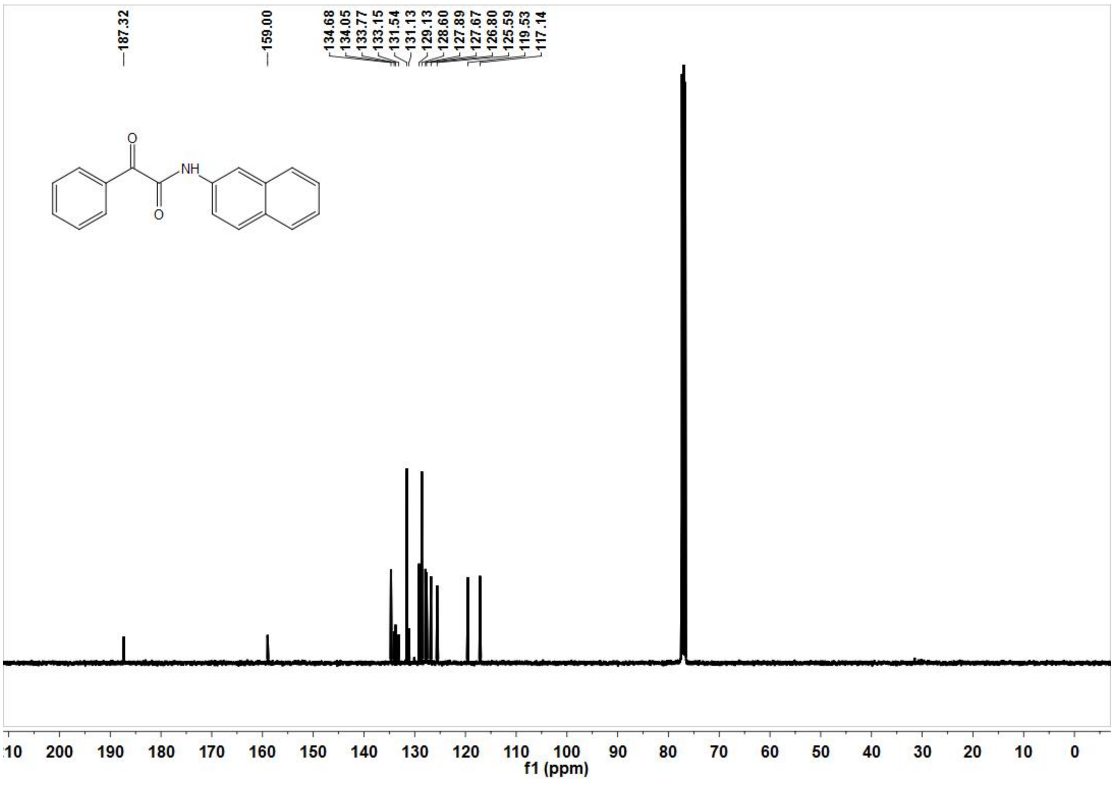

Figure S20. ^{13}C NMR (100 MHz, $CDCl_3$) spectrum of **4j.**

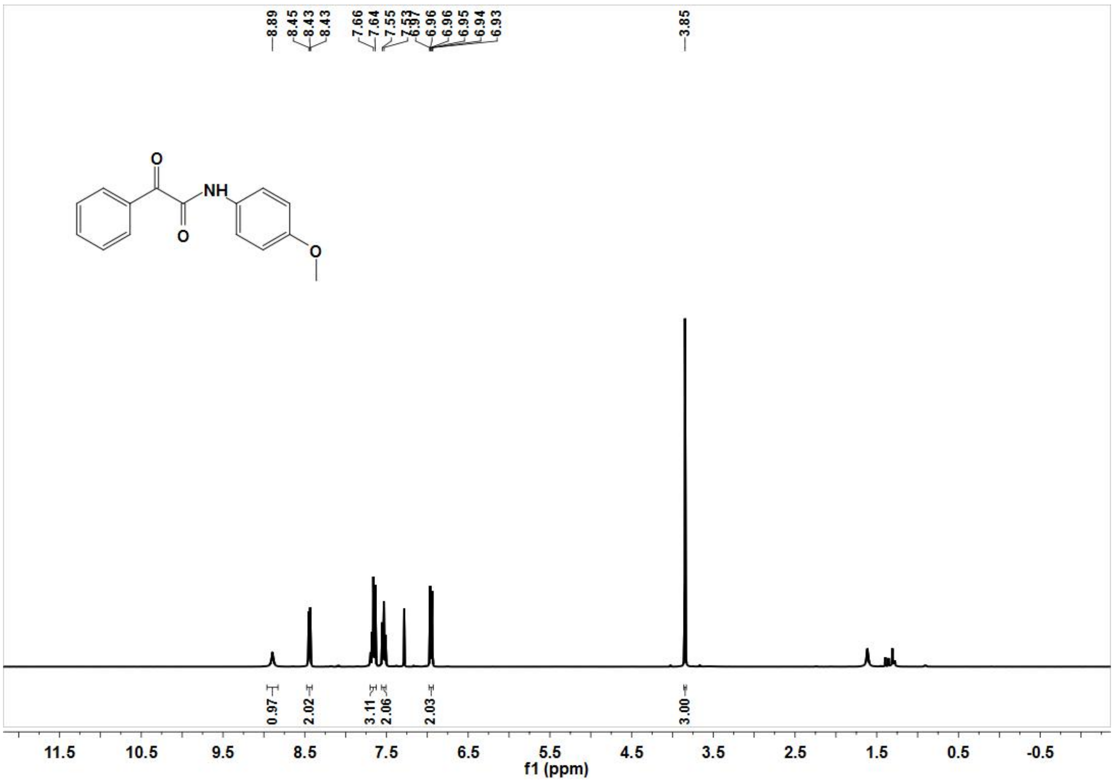

Figure S21. ^{1}H NMR (400 MHz, $CDCl_3$) spectrum of **4k.**

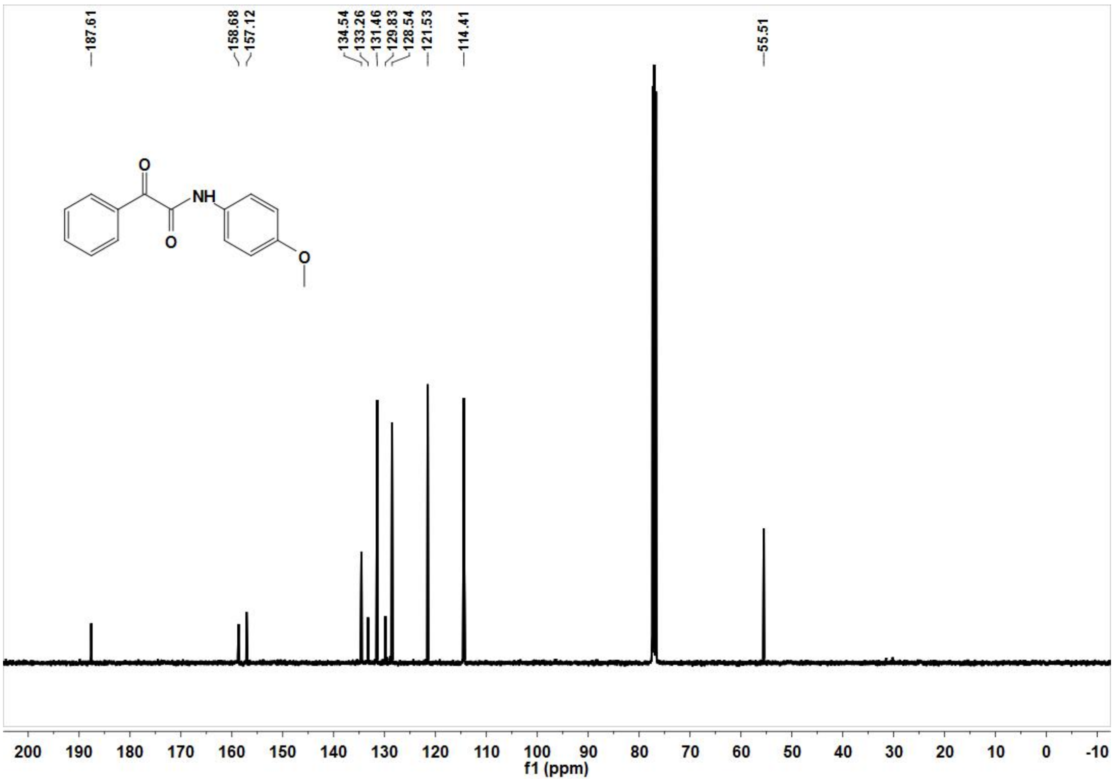

Figure S22. ^{13}C NMR (100 MHz, $CDCl_3$) spectrum of **4k.**

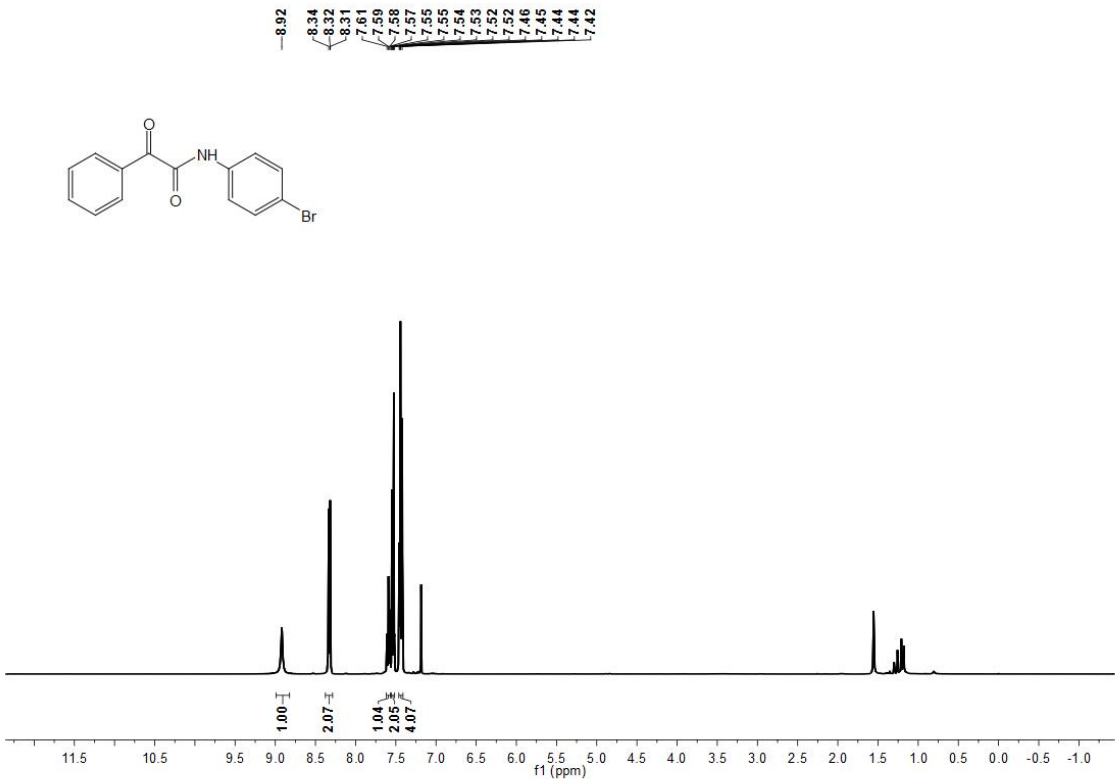

Figure S23. ^{1}H NMR (400 MHz, $CDCl_3$) spectrum of **4l.**

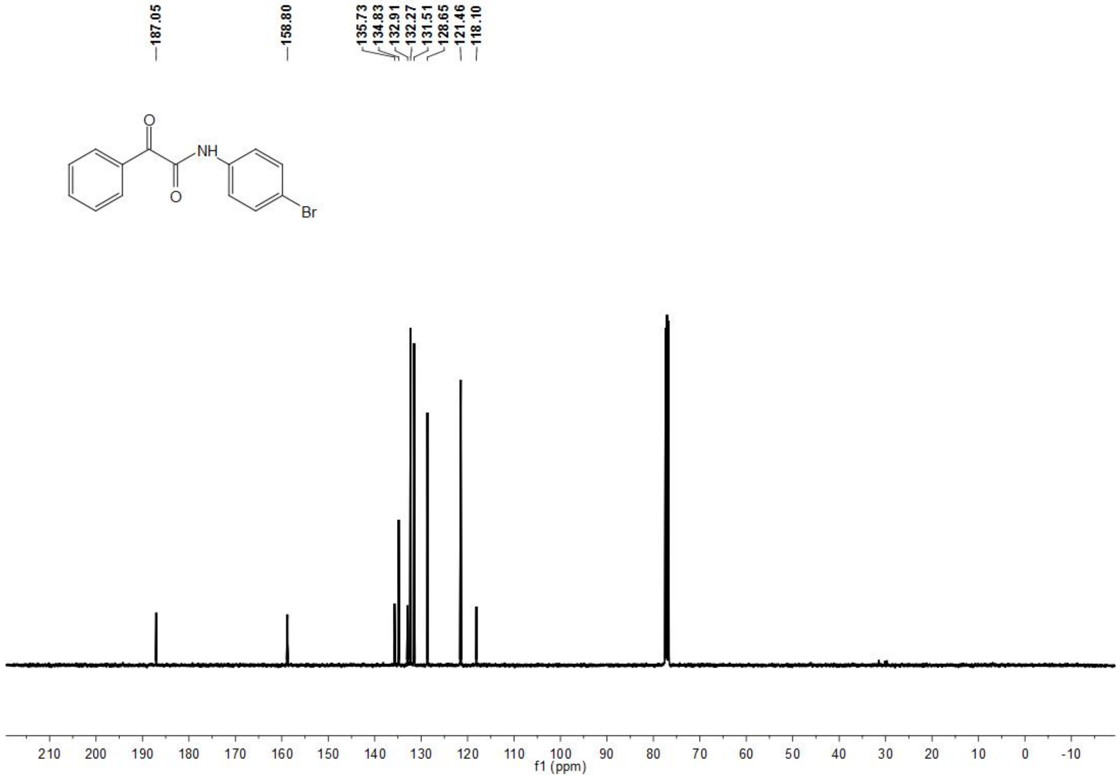

Figure S24. ^{13}C NMR (100 MHz, $CDCl_3$) spectrum of **4l.**

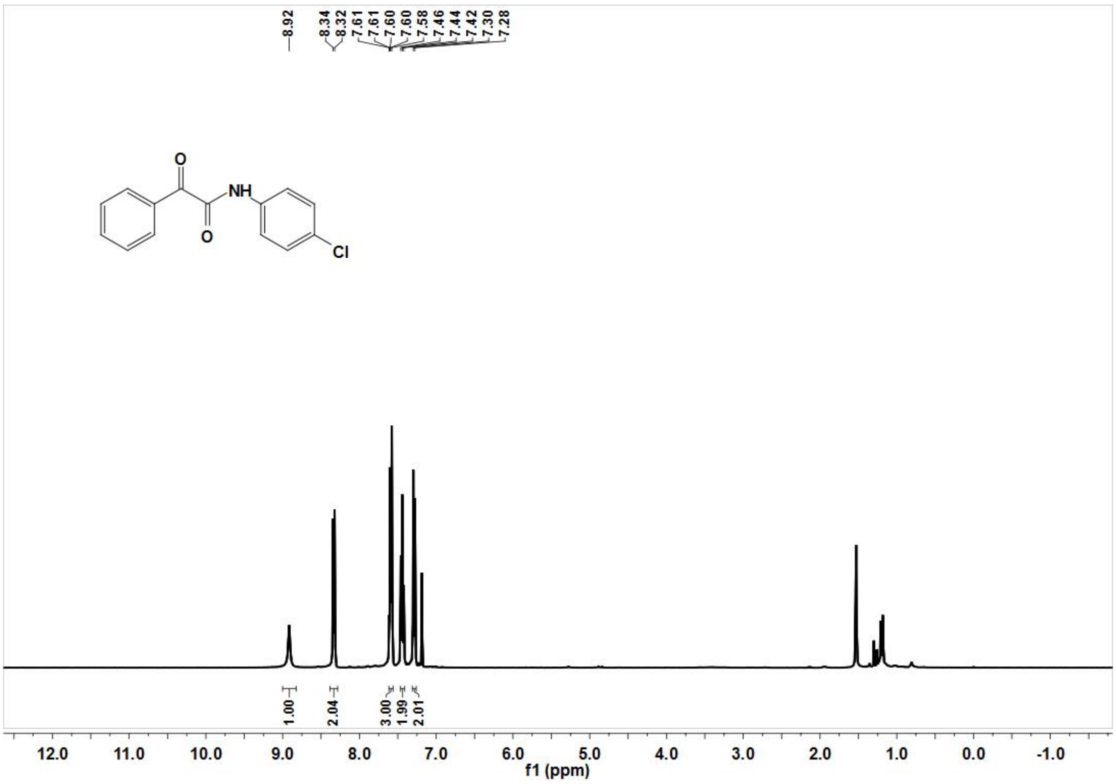

Figure S25. ^{1}H NMR (400 MHz, $CDCl_3$) spectrum of **4m.**

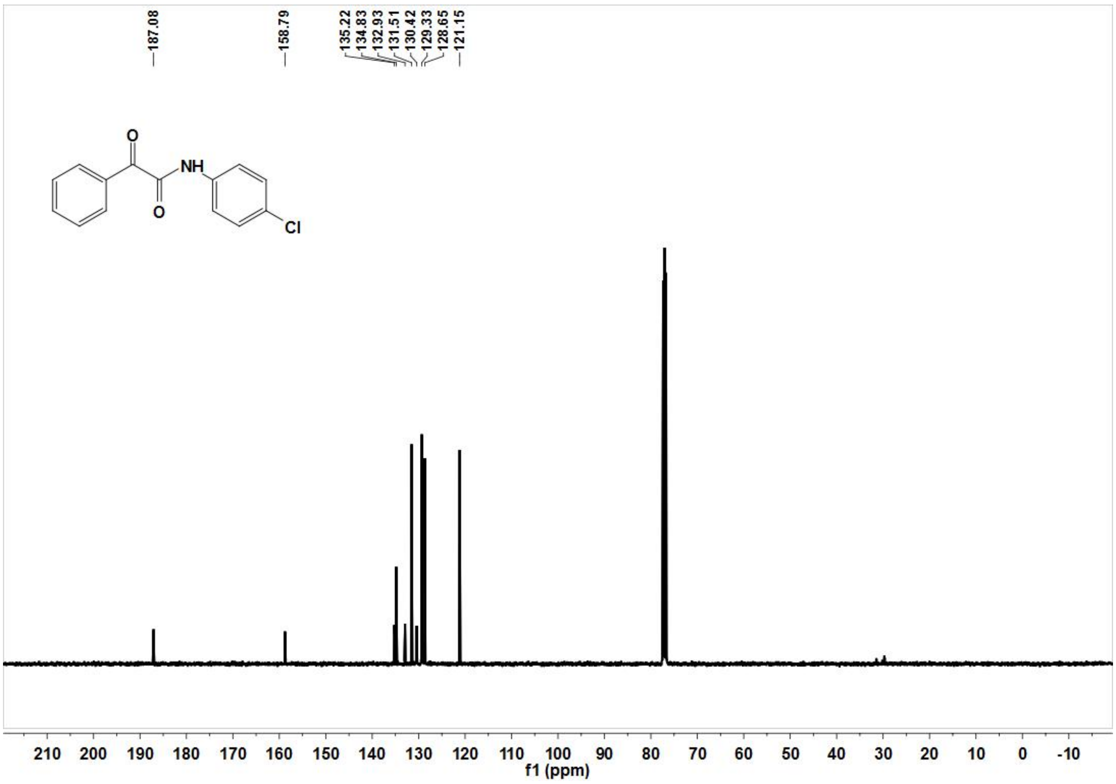

Figure S26. ^{13}C NMR (100 MHz, $CDCl_3$) spectrum of **4m.**

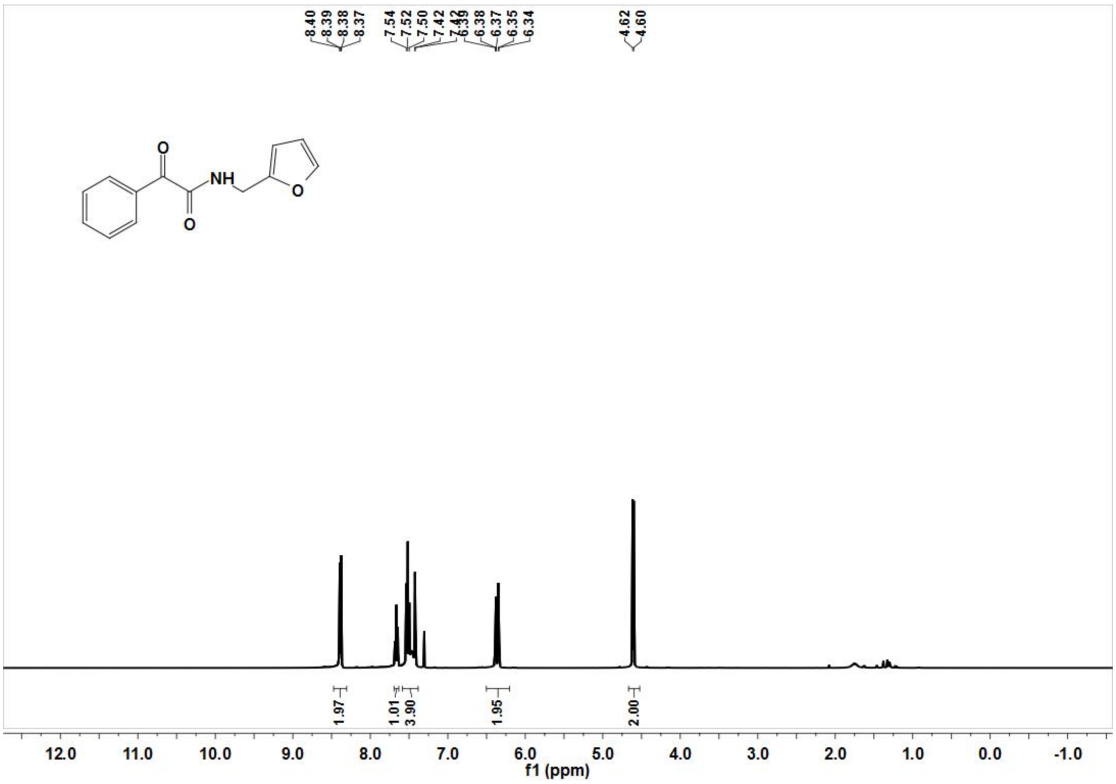

Figure S27. ^{1}H NMR (400 MHz, $CDCl_3$) spectrum of **4n.**

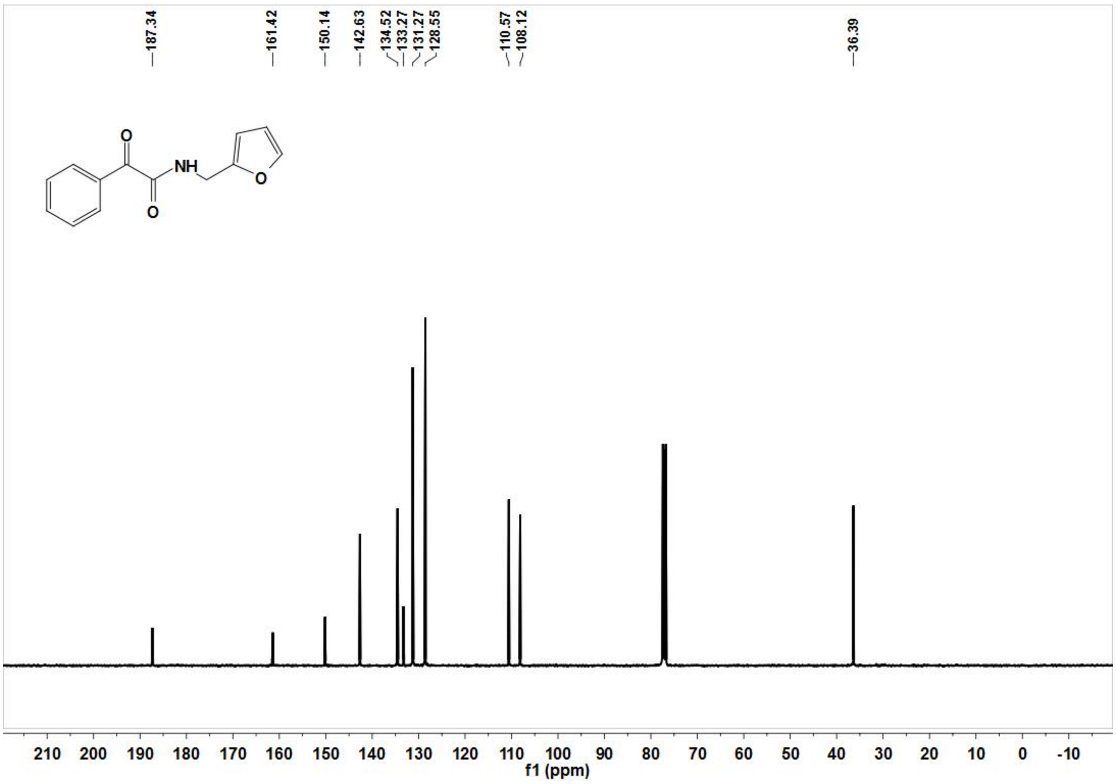

Figure S28. ^{13}C NMR (100 MHz, $CDCl_3$) spectrum of **4n.**

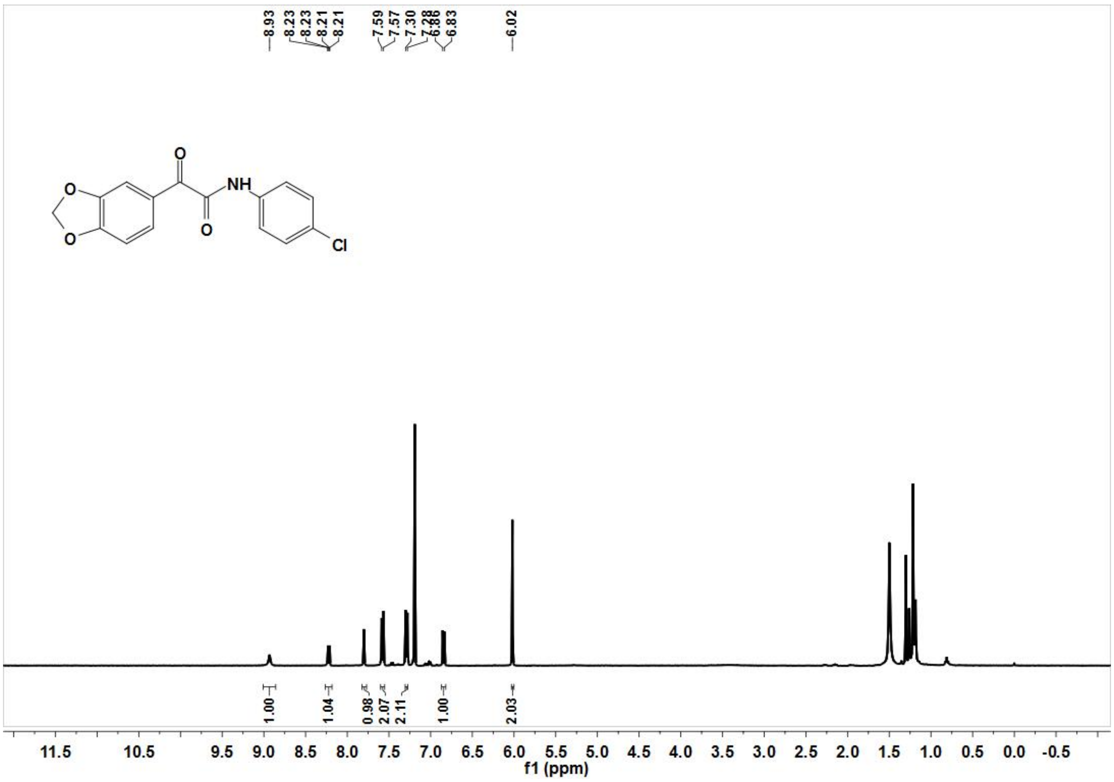

Figure S29. ^{1}H NMR (400 MHz, $CDCl_3$) spectrum of **4o.**

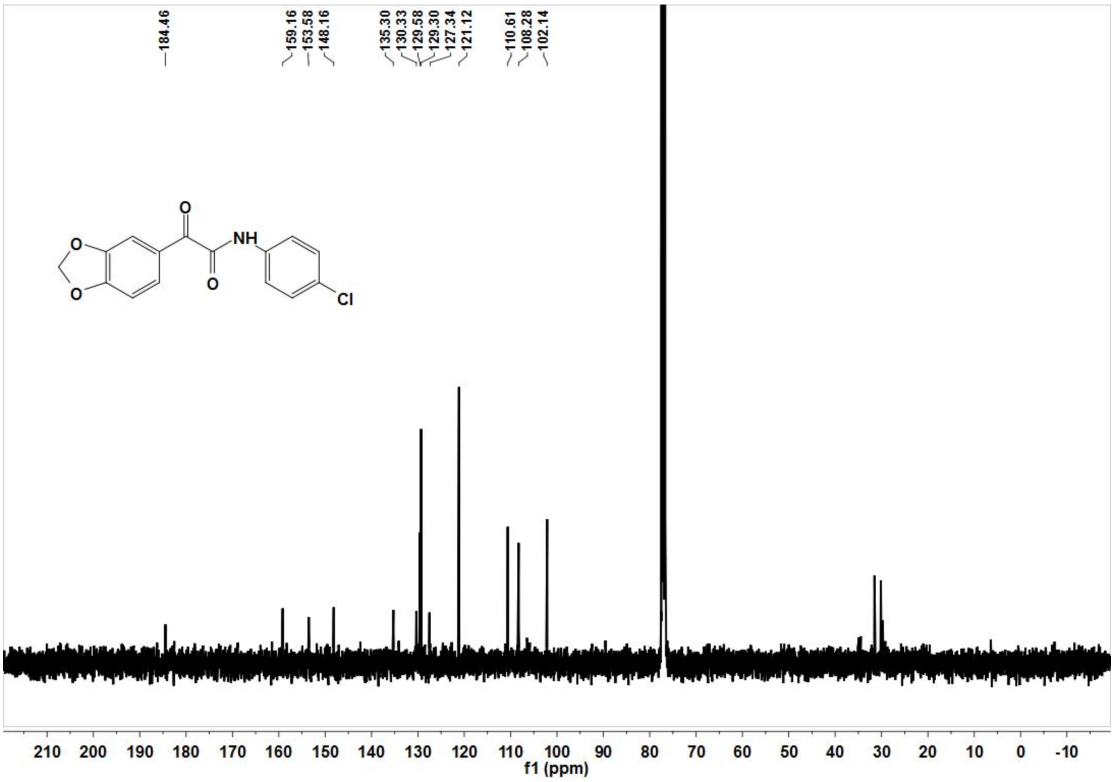

Figure S30. ^{13}C NMR (100 MHz, $CDCl_3$) spectrum of **4o.**

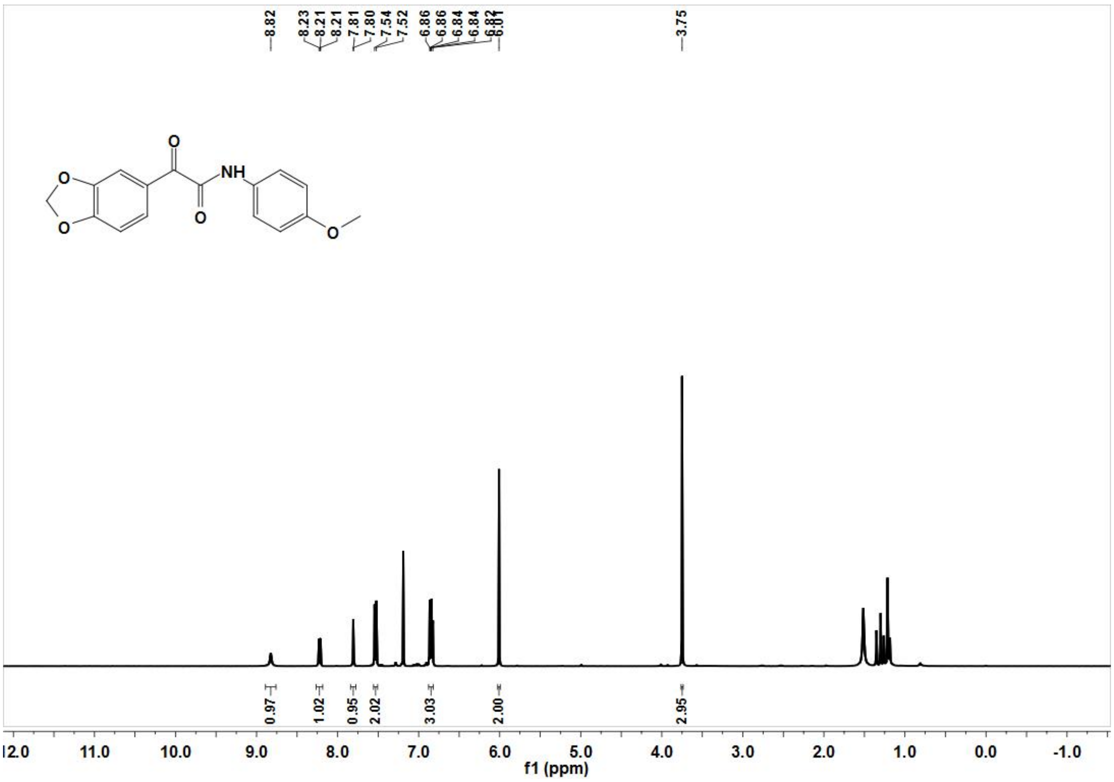

Figure S31. ^{1}H NMR (400 MHz, $CDCl_3$) spectrum of **4p.**

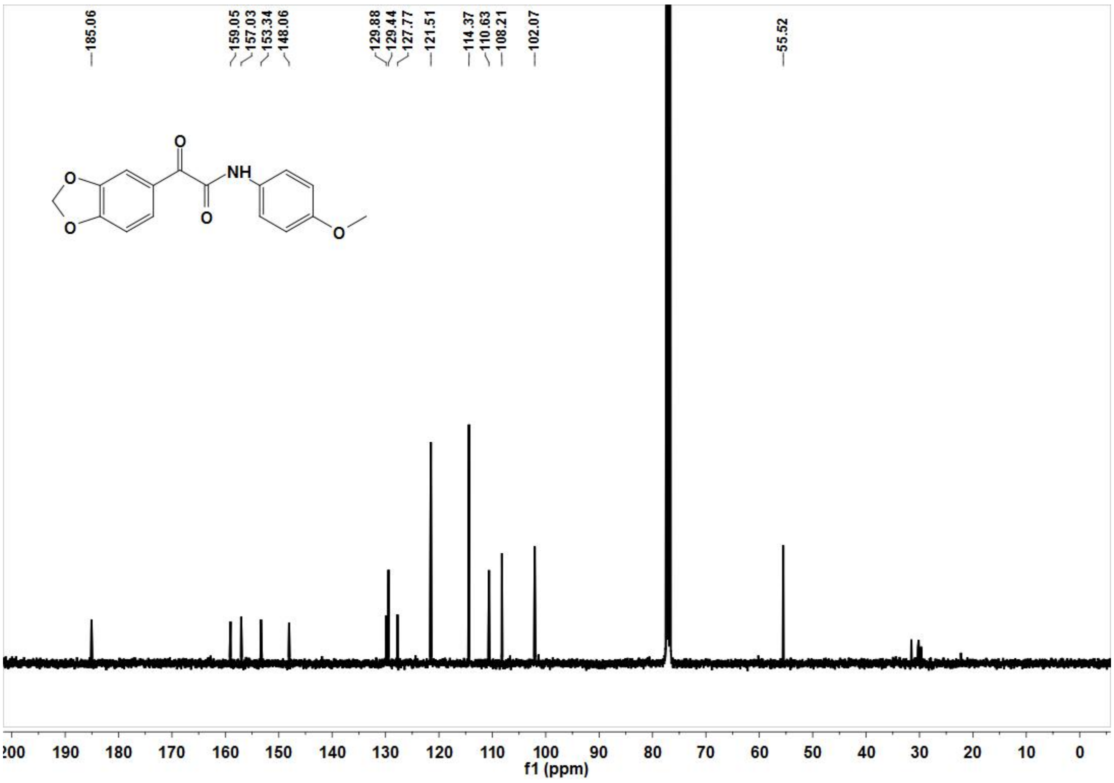

Figure S32. ^{13}C NMR (100 MHz, $CDCl_3$) spectrum of **4p.**

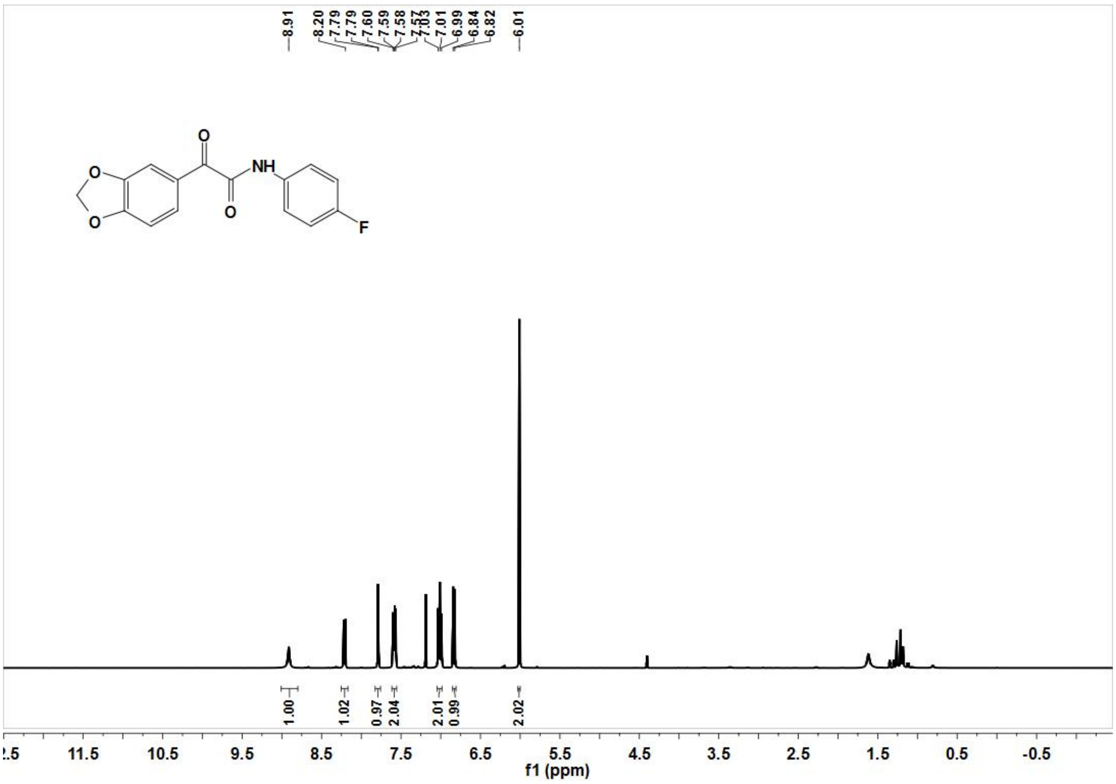

Figure S33. ^{1}H NMR (400 MHz, $CDCl_3$) spectrum of **4q.**

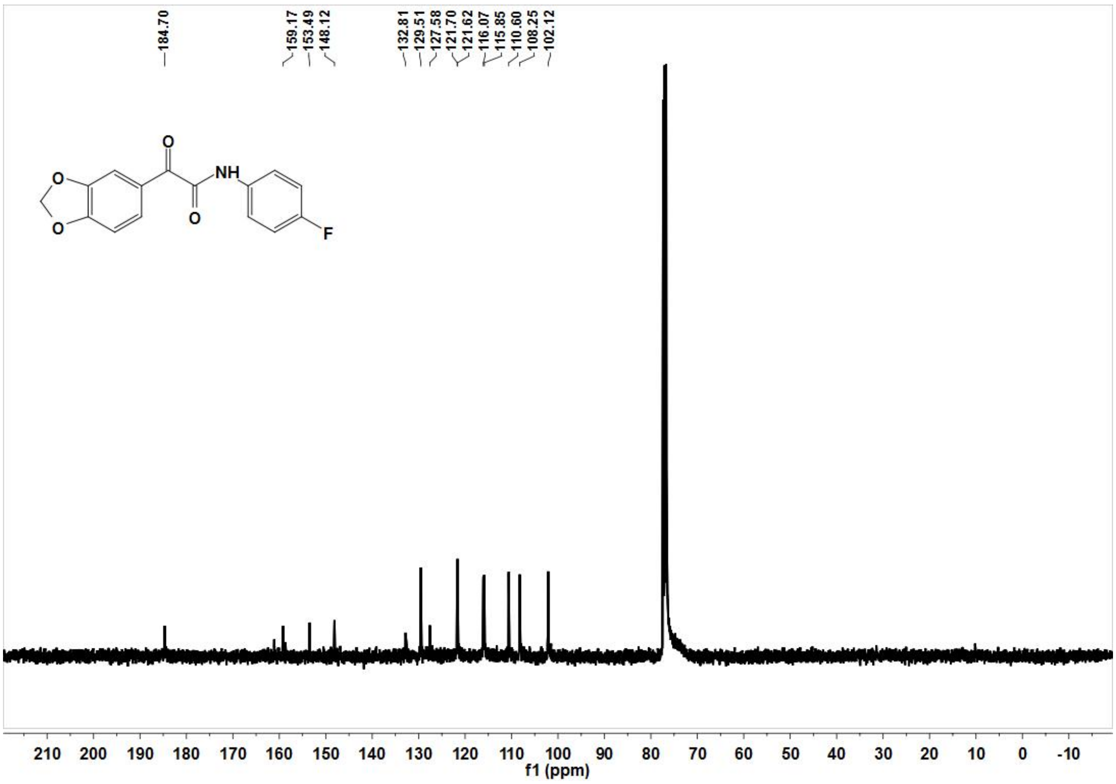

Figure S34. ^{13}C NMR (100 MHz, $CDCl_3$) spectrum of **4q.**

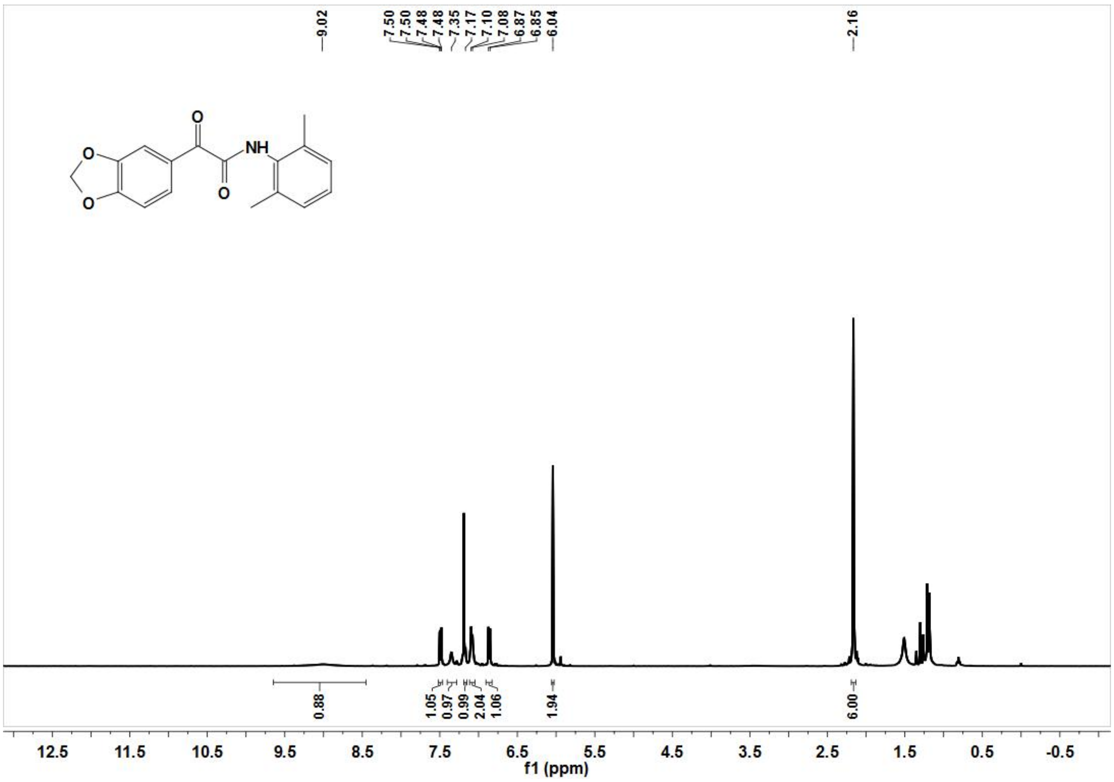

Figure S35. ^{1}H NMR (400 MHz, $CDCl_3$) spectrum of **4r.**

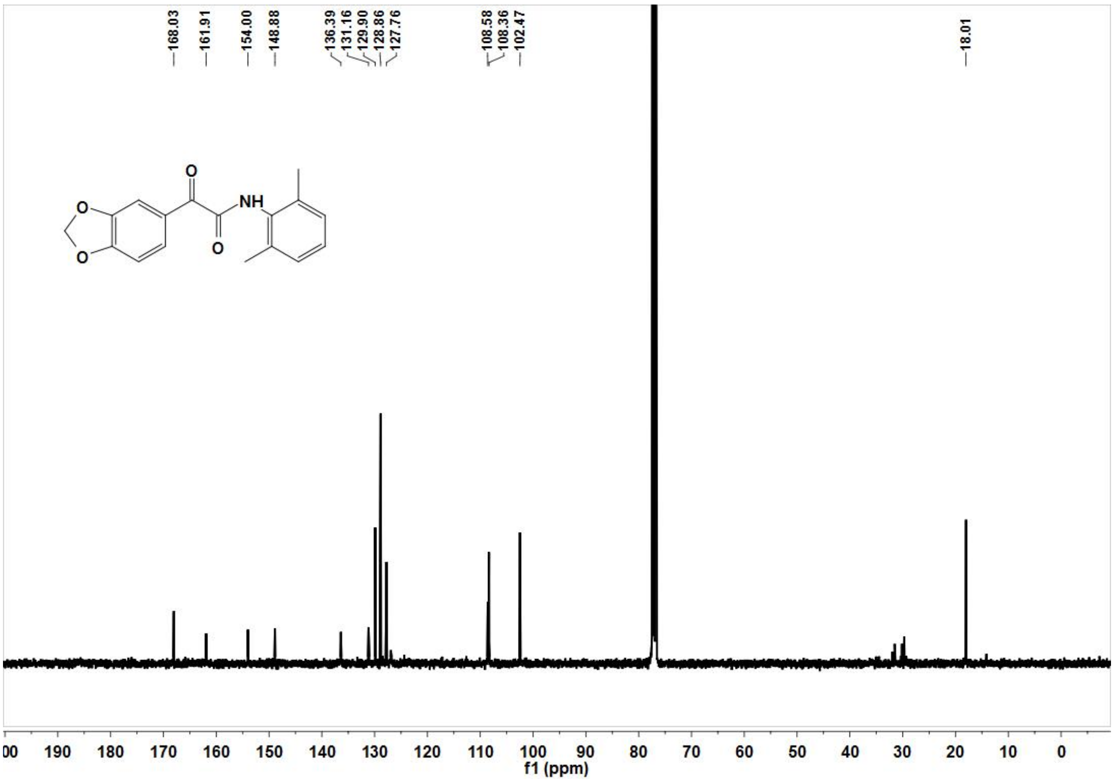

Figure S36. ^{13}C NMR (100 MHz, $CDCl_3$) spectrum of **4r.**

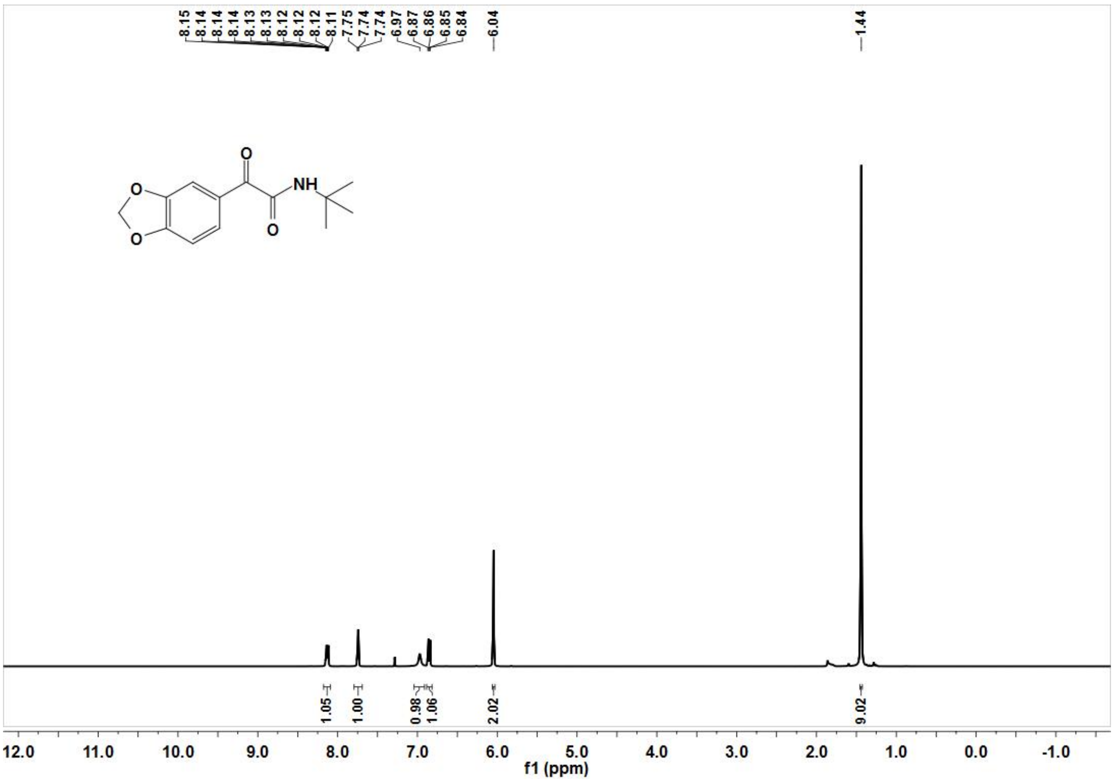

Figure S37. ^{1}H NMR (400 MHz, $CDCl_3$) spectrum of **4s.**

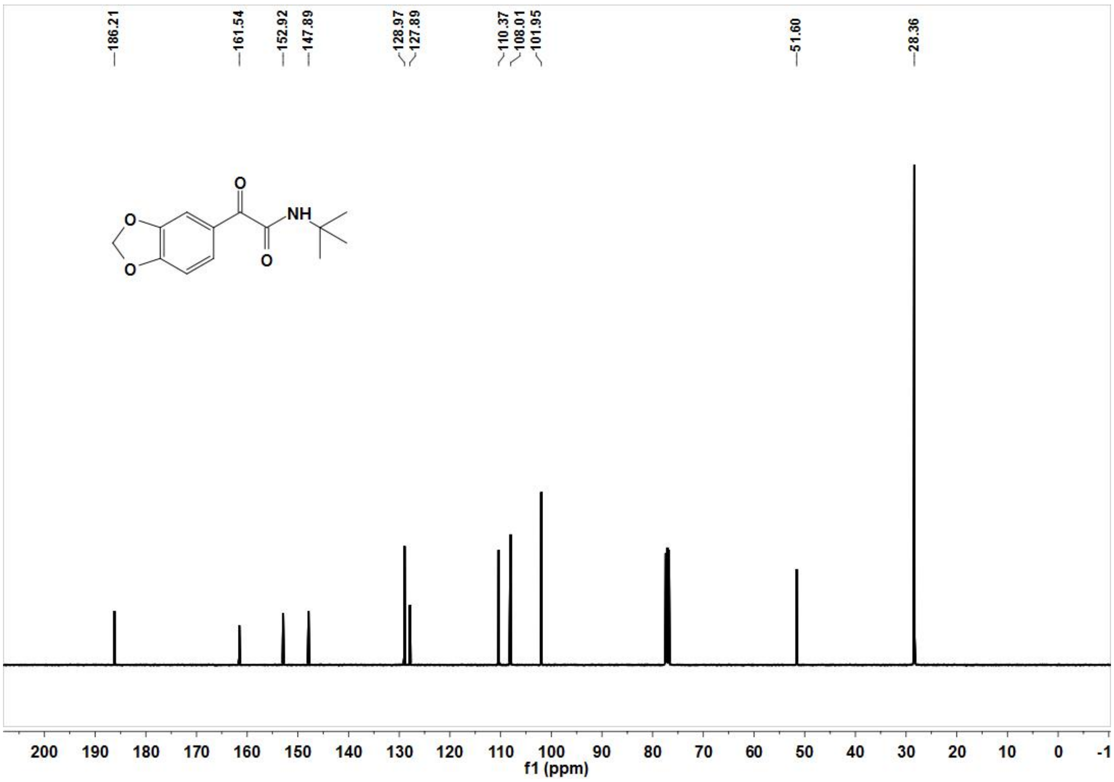

Figure S38. ^{13}C NMR (100 MHz, $CDCl_3$) spectrum of **4s.**

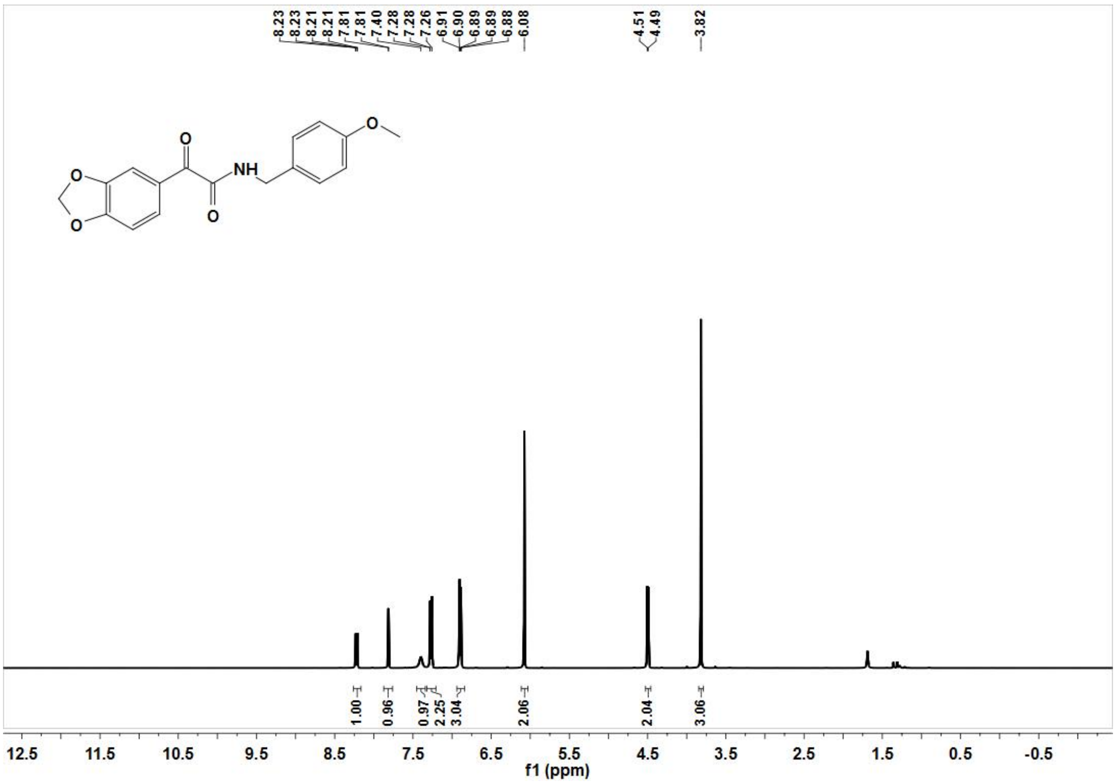

Figure S39. ^{1}H NMR (400 MHz, $CDCl_3$) spectrum of **4t.**

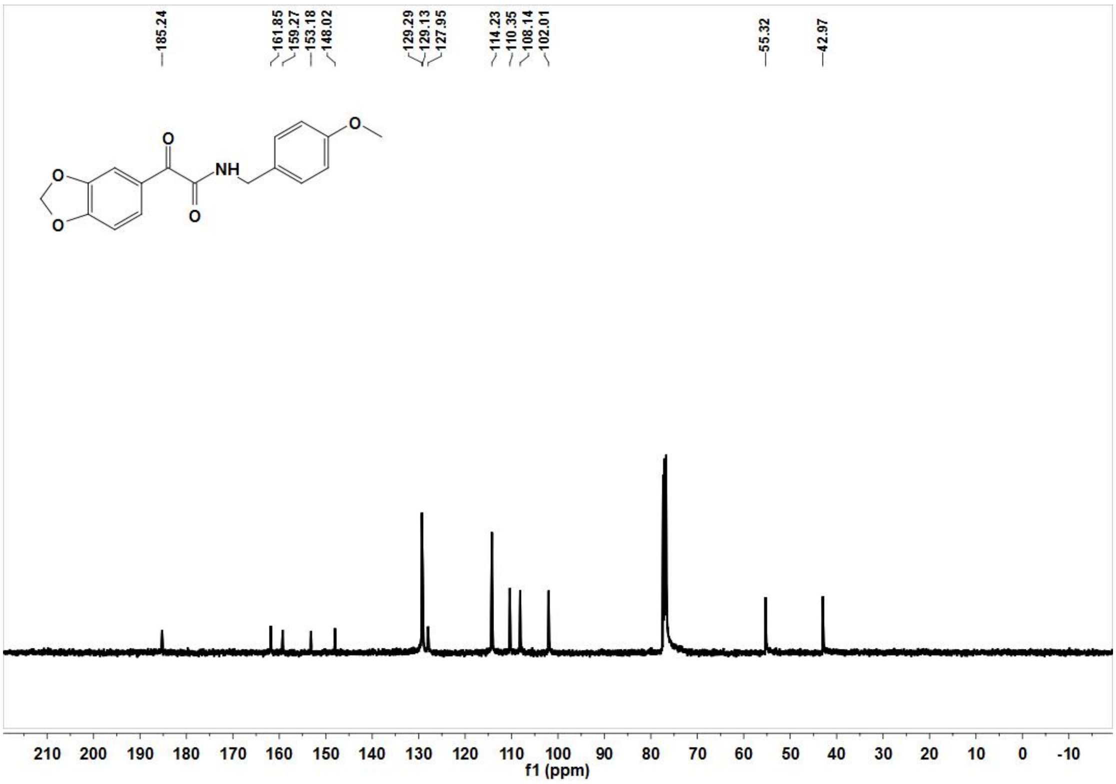

Figure S40. ^{13}C NMR (100 MHz, $CDCl_3$) spectrum of **4t.**

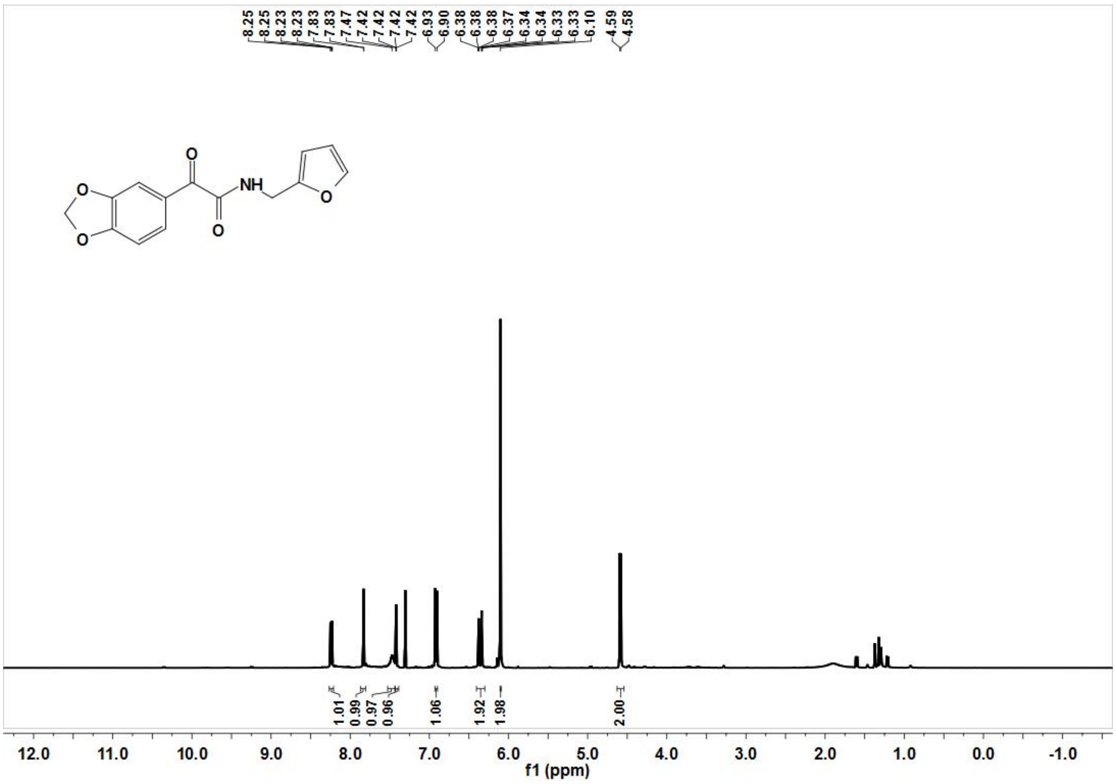

Figure S41. ^{1}H NMR (400 MHz, $CDCl_3$) spectrum of **4u.**

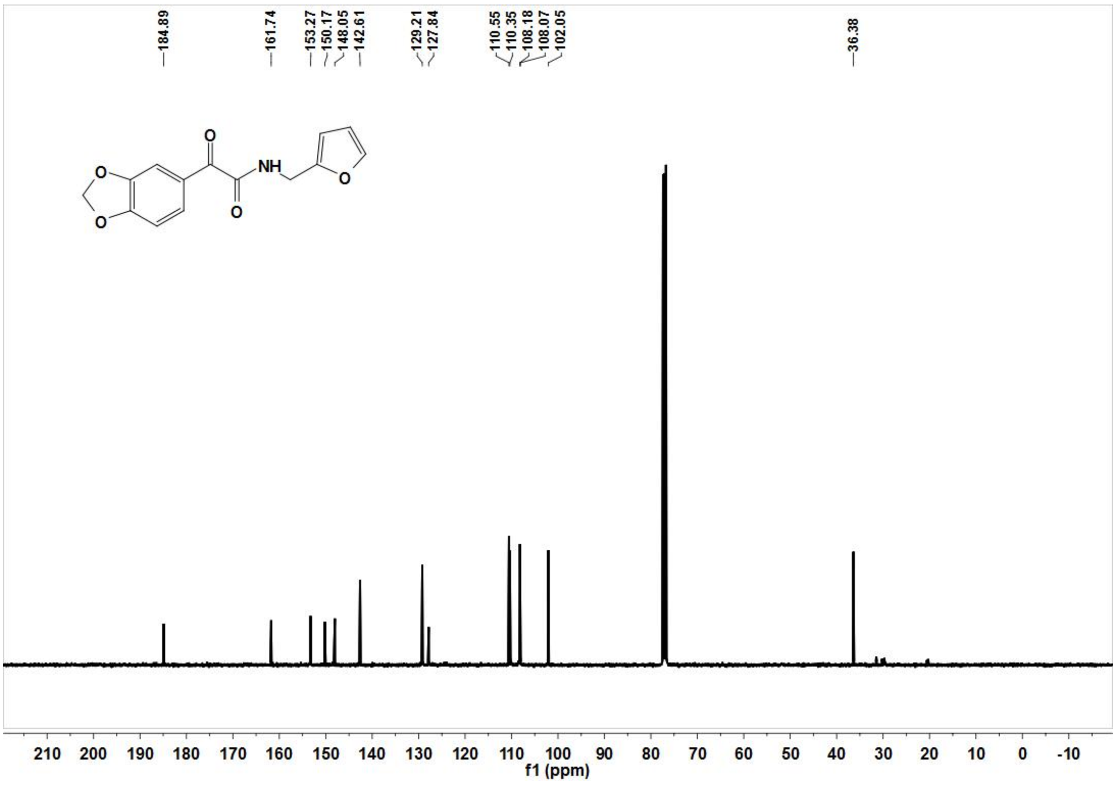

Figure S42. ^{13}C NMR (100 MHz, $CDCl_3$) spectrum of **4u.**

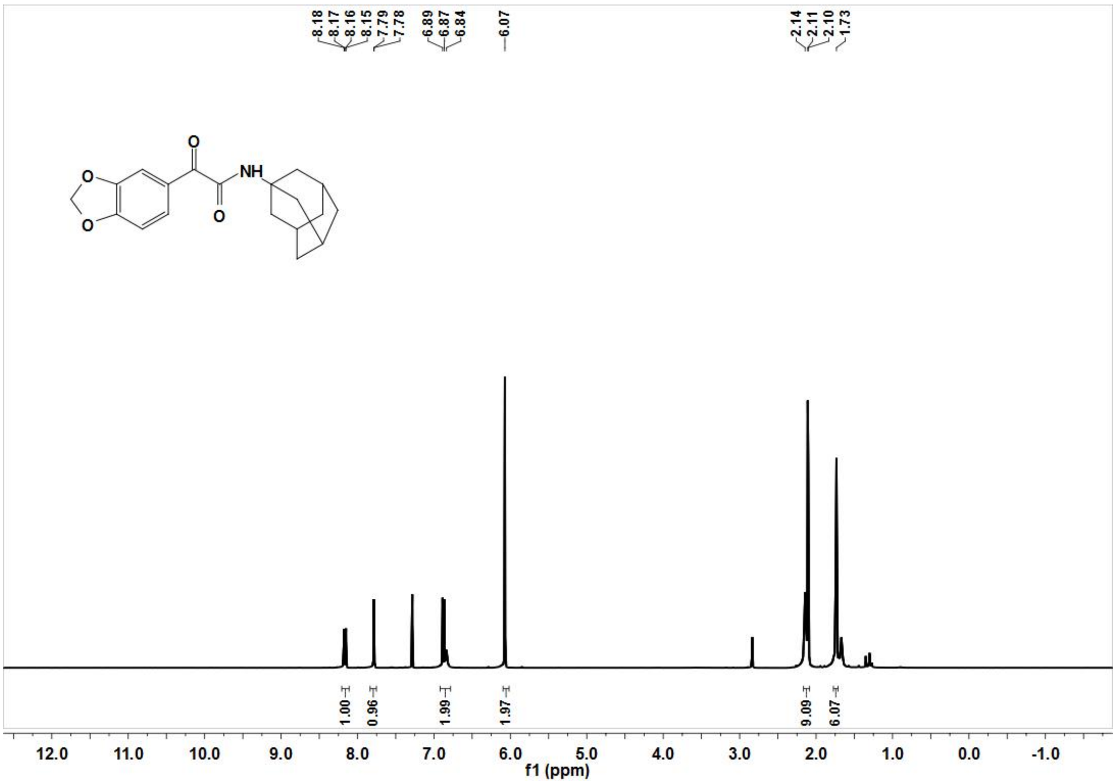

Figure S43. ^{1}H NMR (400 MHz, $CDCl_3$) spectrum of **4v.**

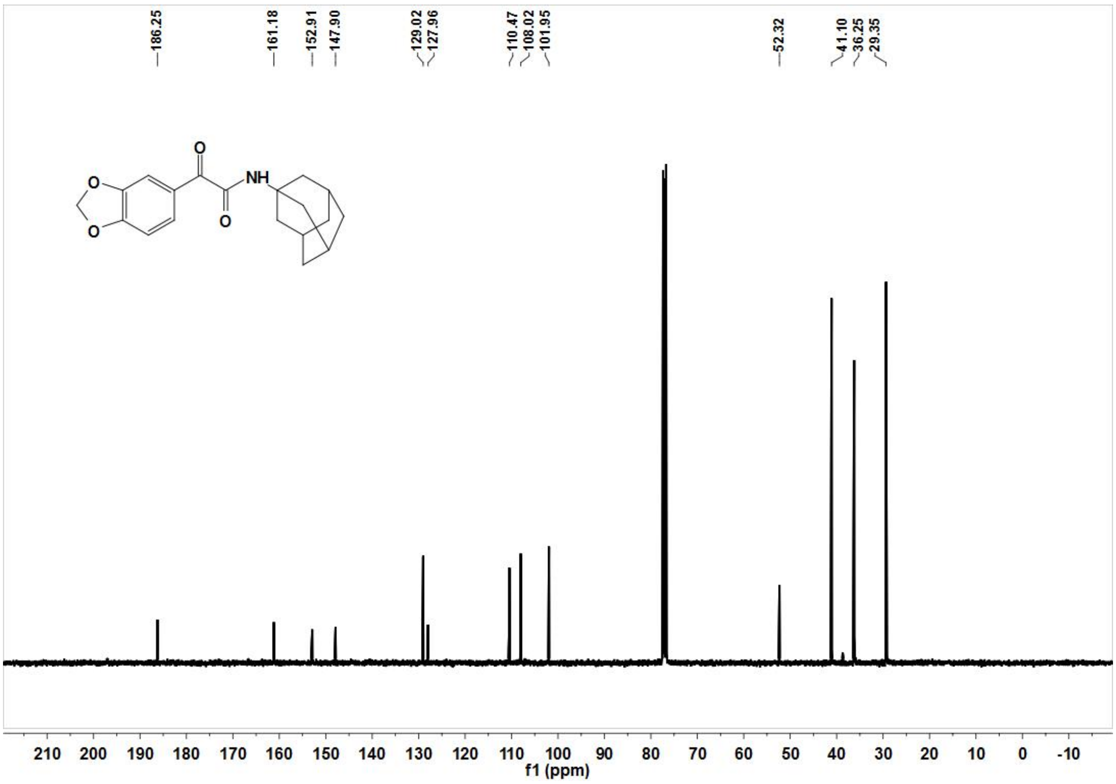

Figure S44. ^{13}C NMR (100 MHz, $CDCl_3$) spectrum of **4v.**

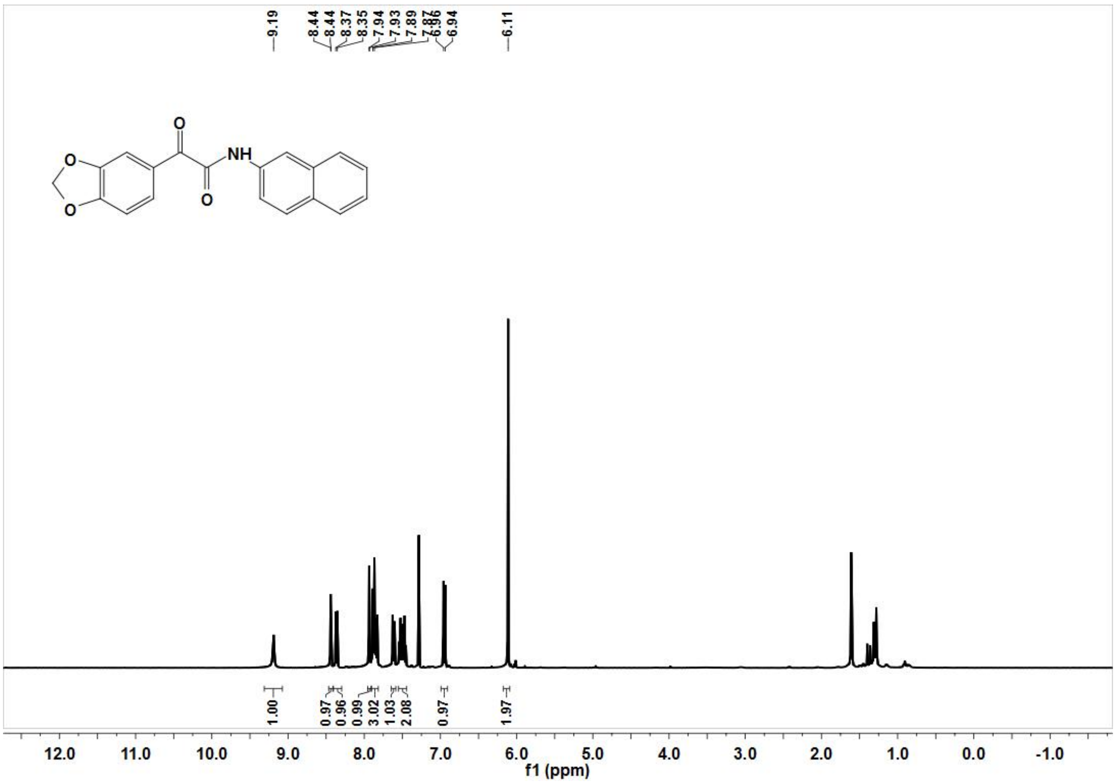

Figure S45. ^{1}H NMR (400 MHz, $CDCl_3$) spectrum of **4w.**

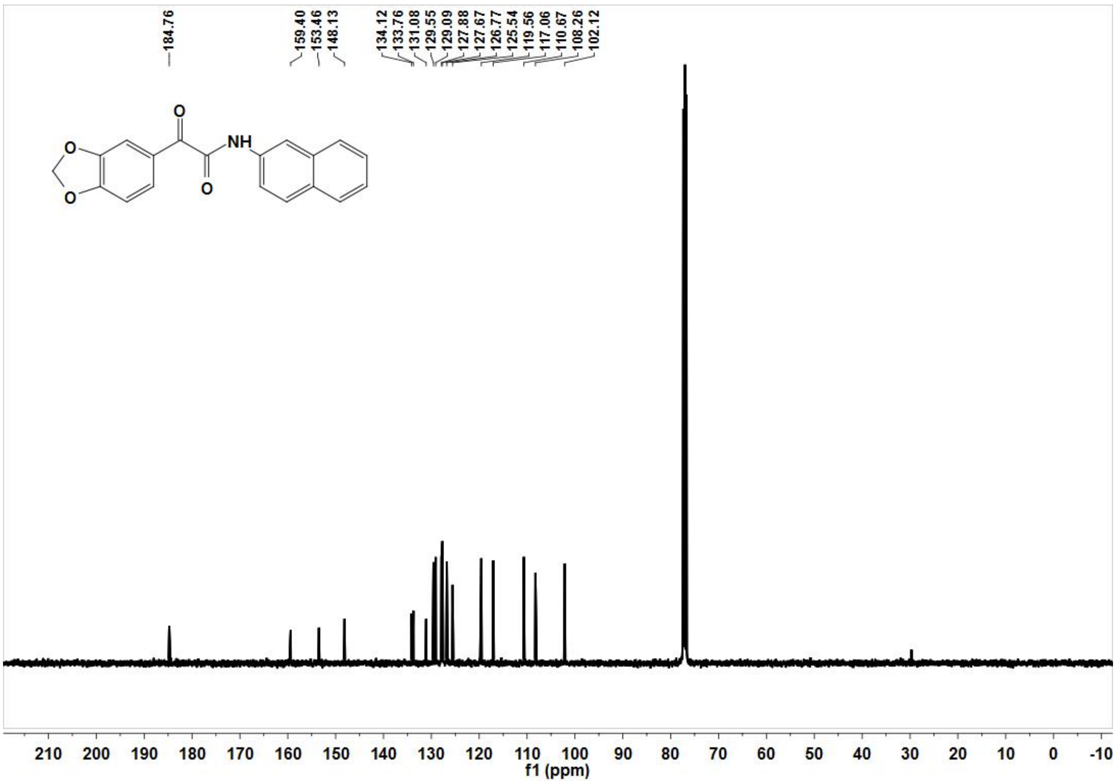

Figure S46. ^{13}C NMR (100 MHz, $CDCl_3$) spectrum of **4w.**

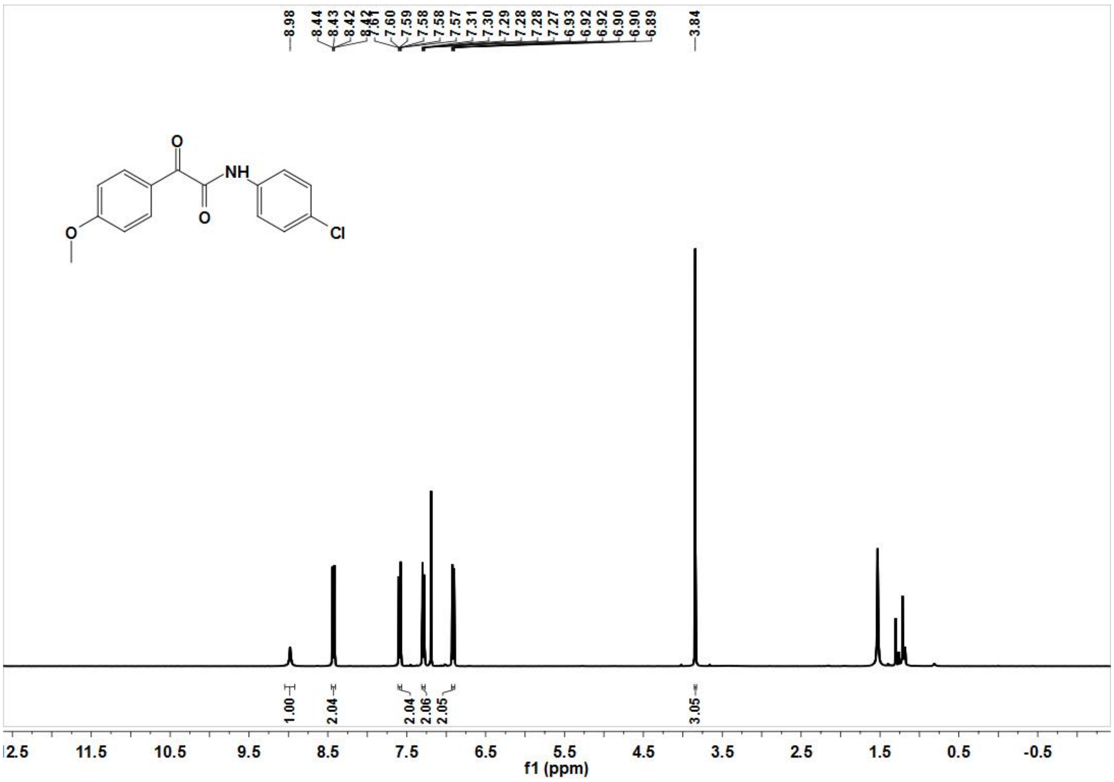

Figure S47. ^{1}H NMR (400 MHz, $CDCl_3$) spectrum of **4x.**

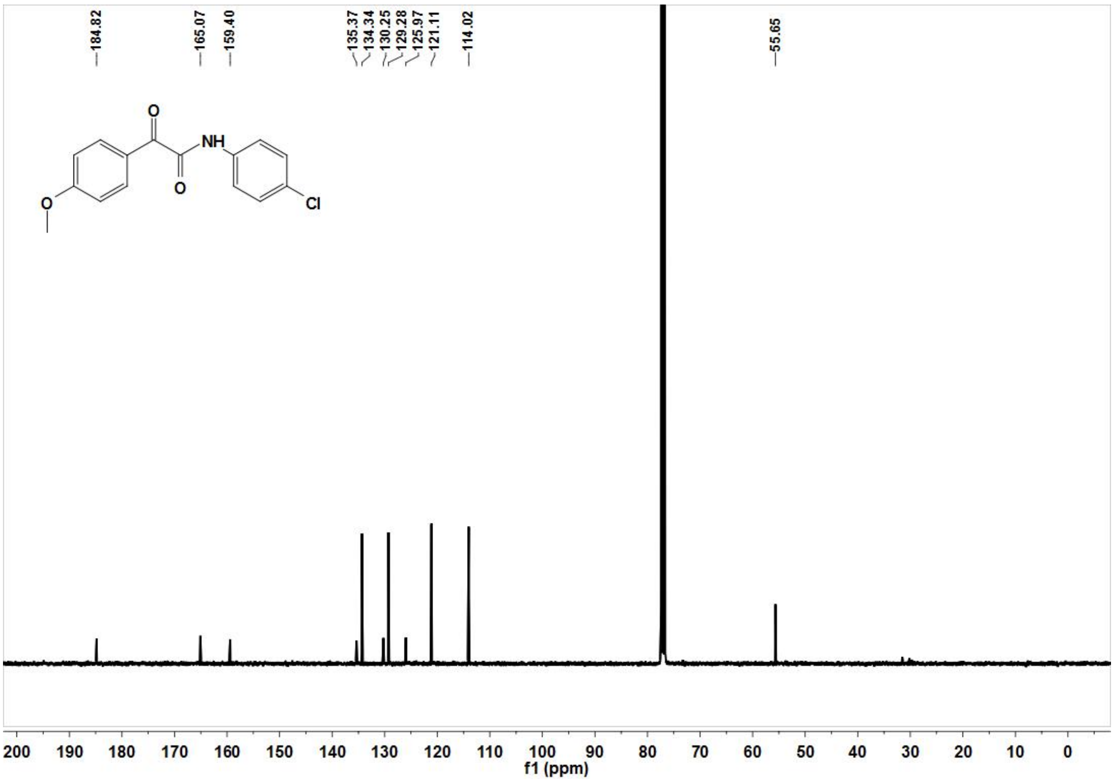

Figure S48. ^{13}C NMR (100 MHz, $CDCl_3$) spectrum of **4x.**

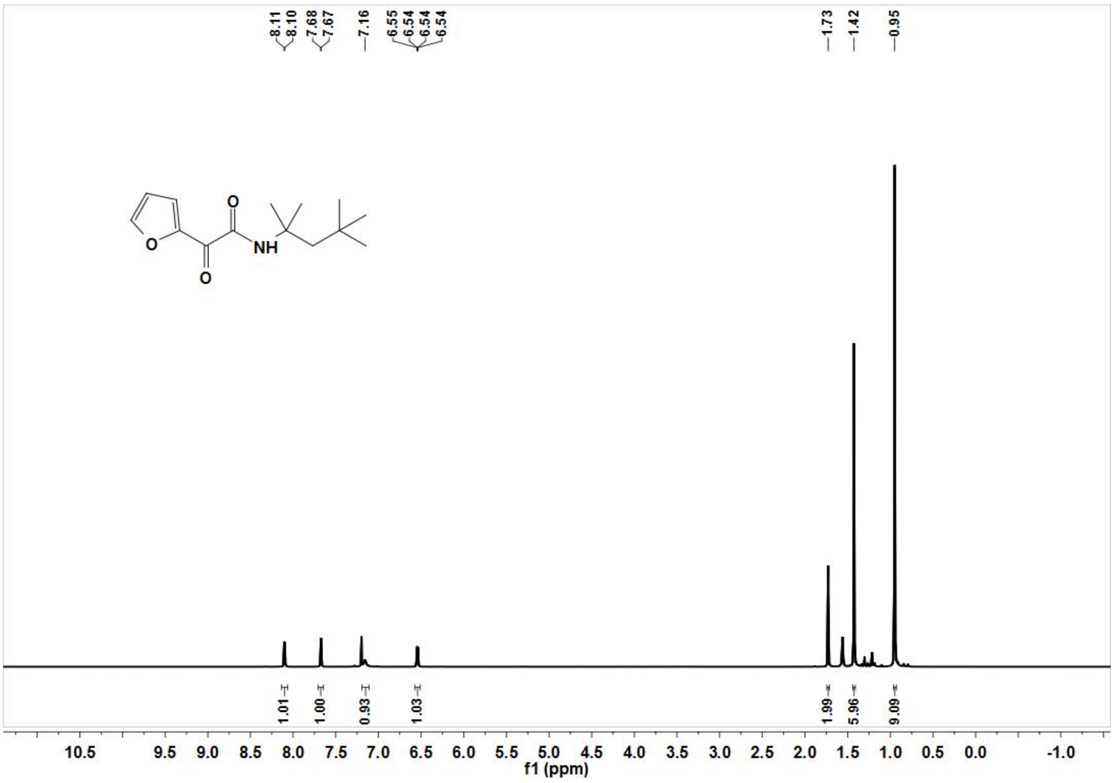

Figure S49. ^{1}H NMR (400 MHz, $CDCl_3$) spectrum of **4y.**

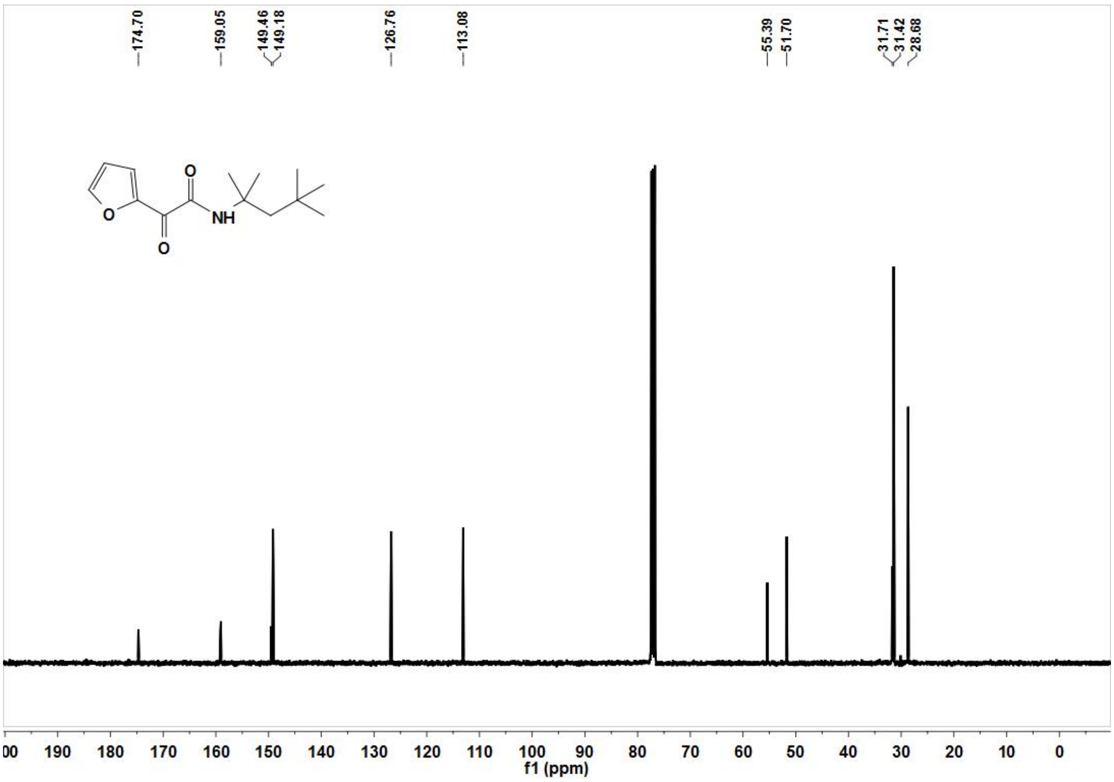

Figure S50. ^{13}C NMR (100 MHz, $CDCl_3$) spectrum of **4y.**

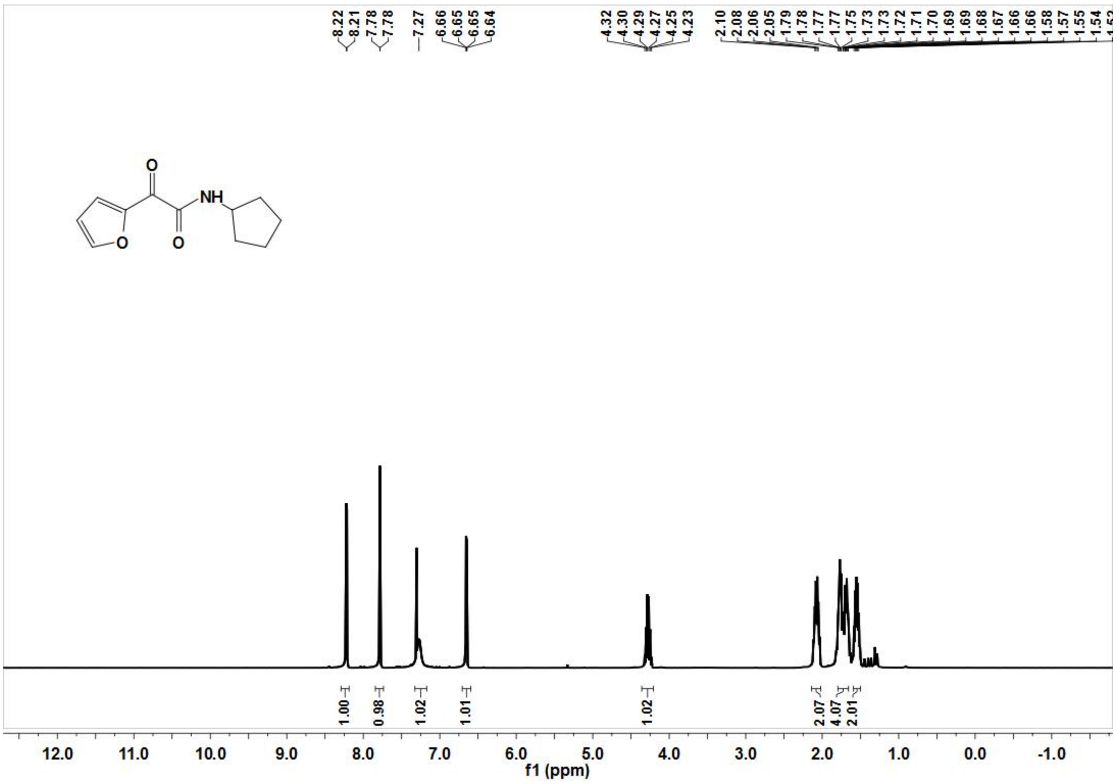

Figure S51. ^{1}H NMR (400 MHz, $CDCl_3$) spectrum of **4z.**

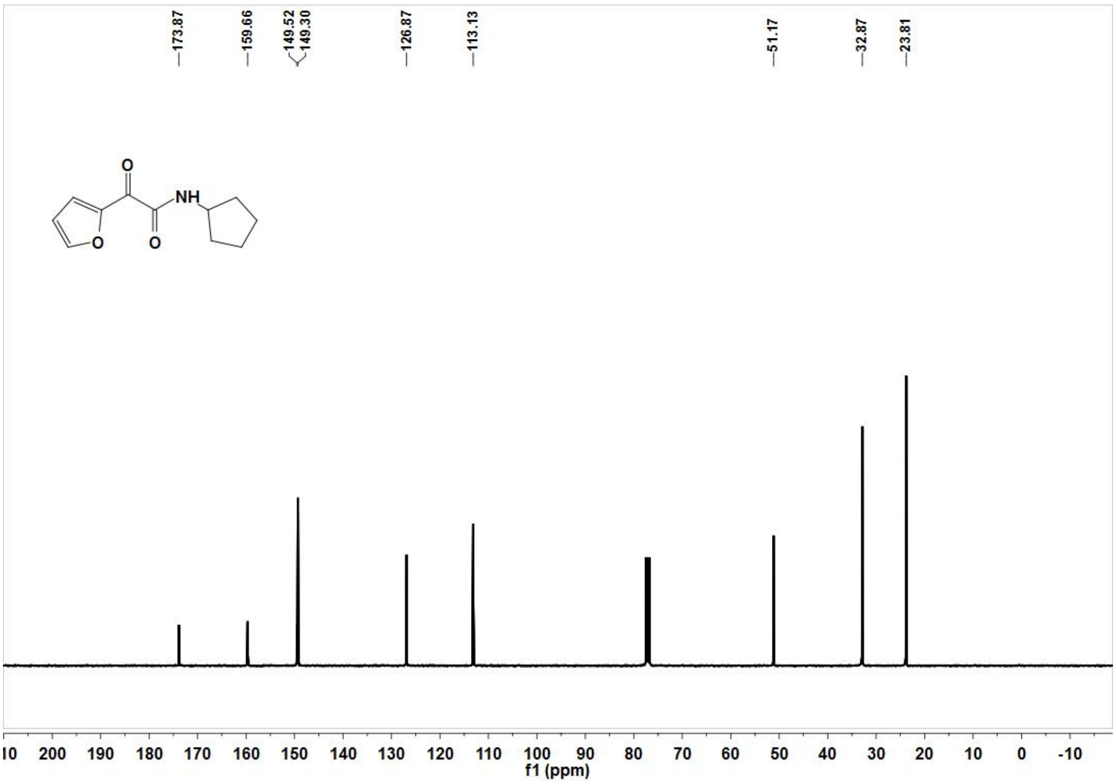

Figure S52. ^{13}C NMR (100 MHz, $CDCl_3$) spectrum of **4z.**

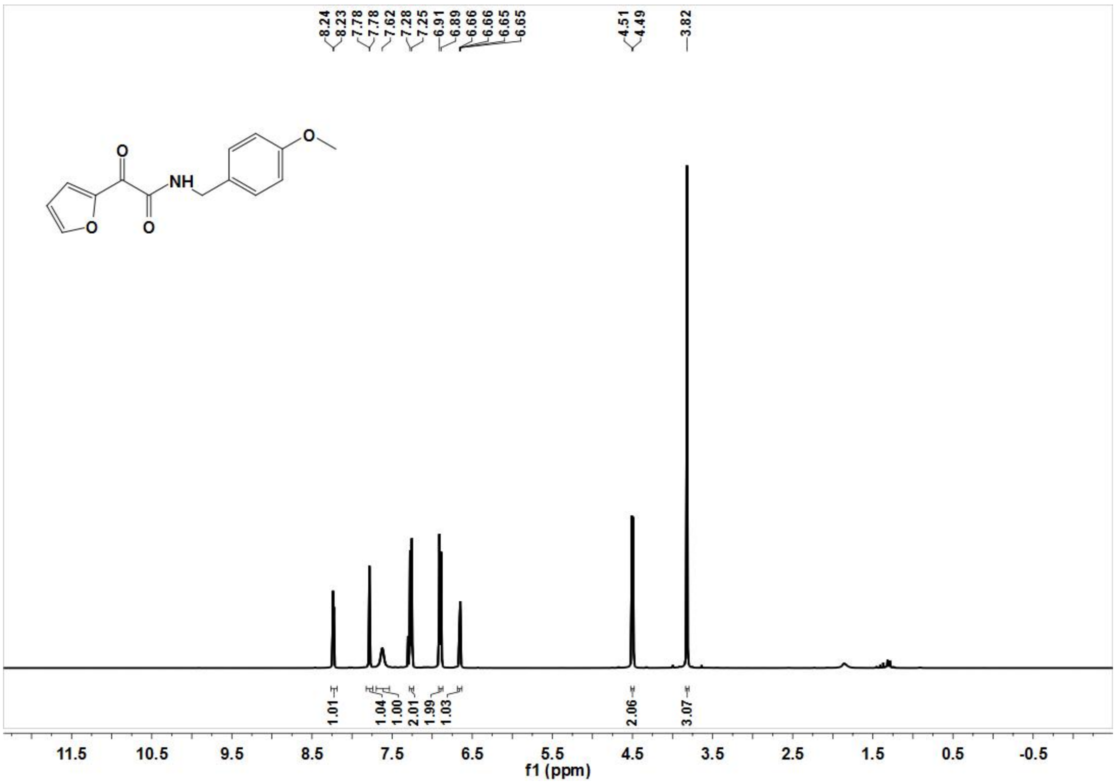

Figure S53. ^{1}H NMR (400 MHz, $CDCl_3$) spectrum of **4aa.**

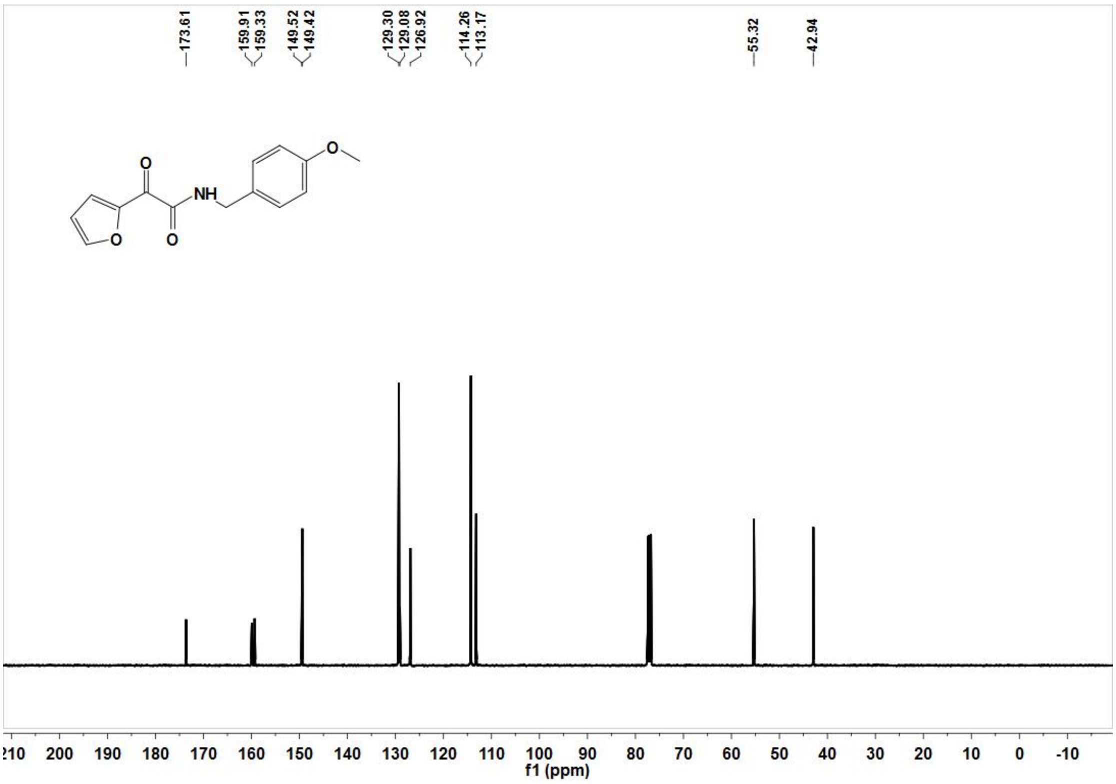

Figure S54. ^{13}C NMR (100 MHz, $CDCl_3$) spectrum of **4aa.**

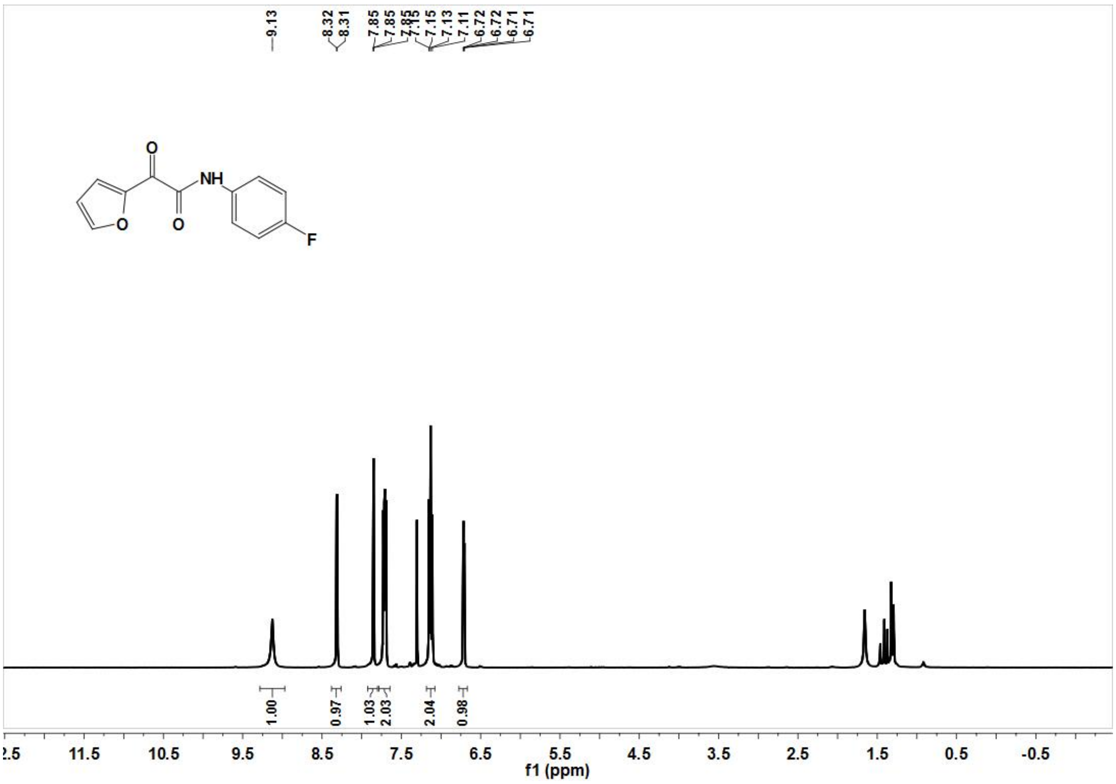

Figure S55. ^{1}H NMR (400 MHz, $CDCl_3$) spectrum of **4ab.**

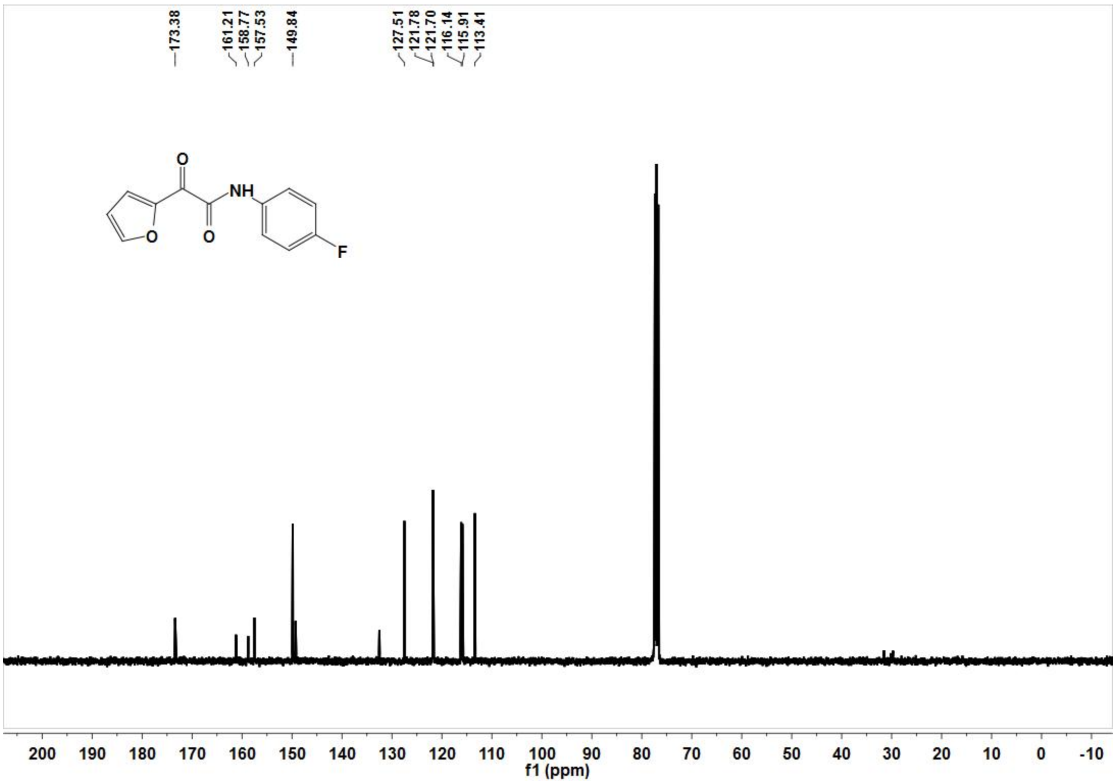

Figure S56. ^{13}C NMR (100 MHz, $CDCl_3$) spectrum of **4ab.**

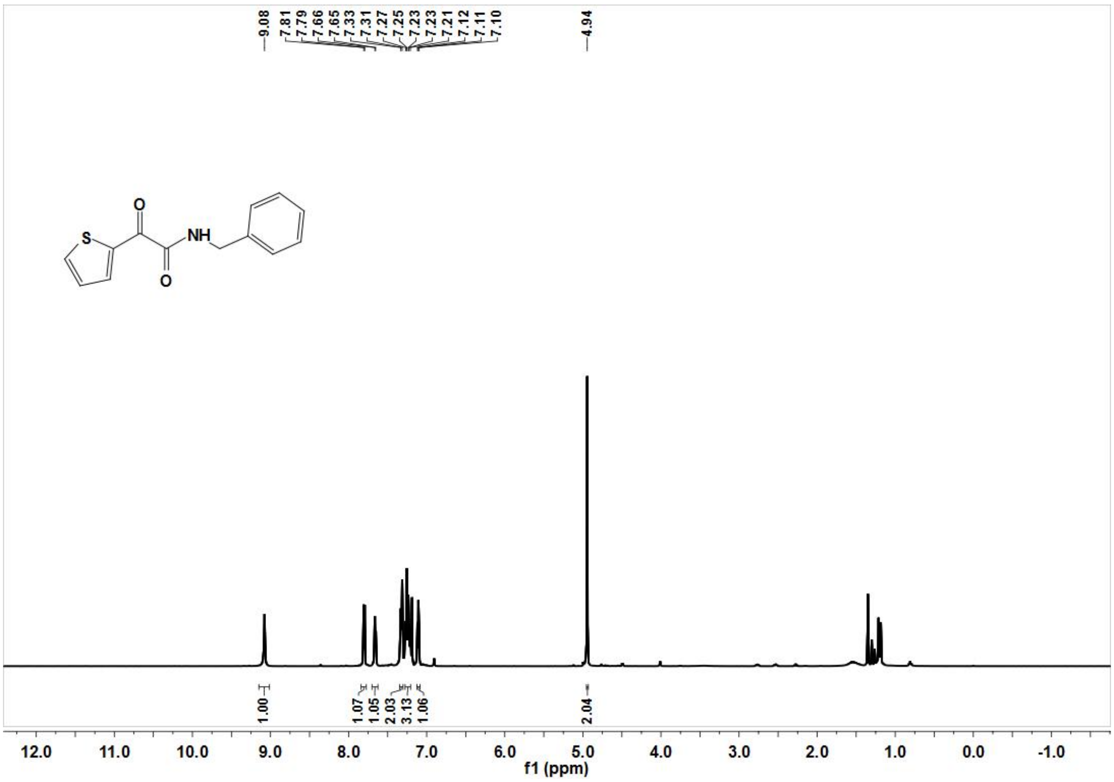

Figure S57. ^{1}H NMR (400 MHz, $CDCl_3$) spectrum of **4ac.**

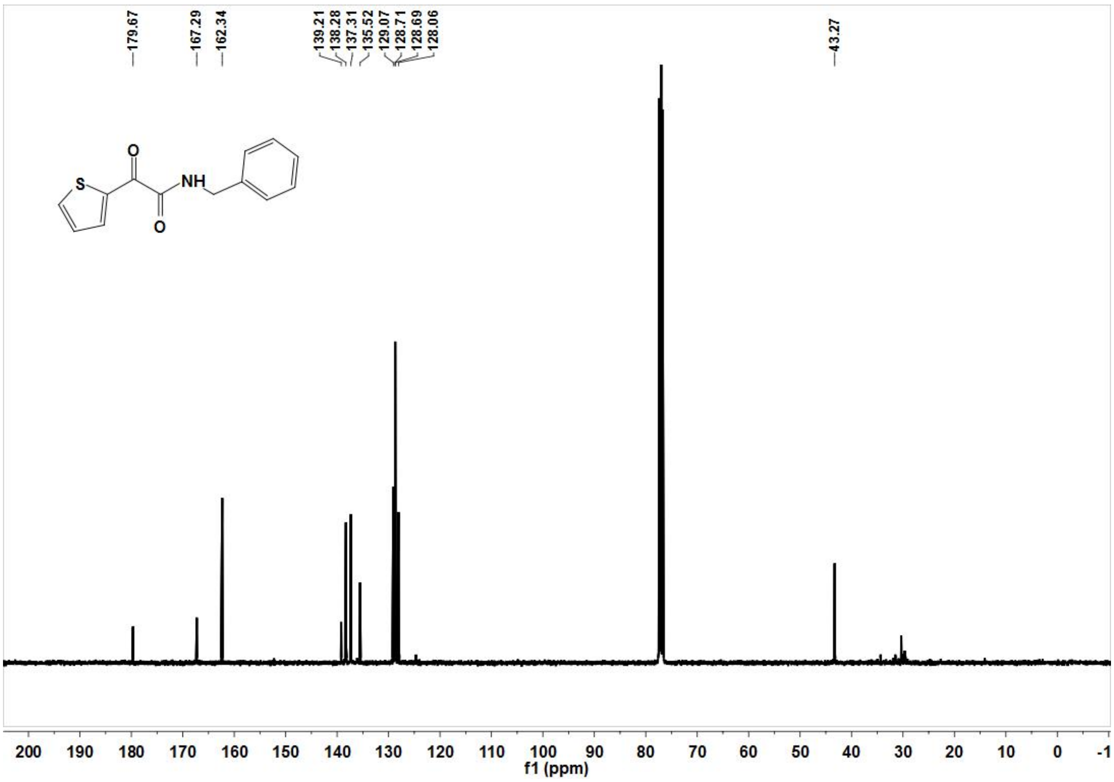

Figure S58. ^{13}C NMR (100 MHz, $CDCl_3$) spectrum of **4ac.**
